# *Conidarnes*, a new oriental genus of Sycophaginae (Hymenoptera, Agaonidae) associated with *Ficus* section *Conosycea* (Moraceae)

**DOI:** 10.3897/zookeys.539.6529

**Published:** 2015-11-23

**Authors:** Fernando Henrique Antoniolli Farache, Jean-Yves Rasplus

**Affiliations:** 1 PPG em Entomologia, Departamento de Biologia/FFCLRP Universidade de São Paulo, Ribeirão Preto, SP, Brazil; 2 INRA, UMR 1062 CBGP Montferrier-sur-Lez, France

**Keywords:** Chalcidoidea, taxonomy, fig, mutualism, non-pollinating fig wasp, gall maker

## Abstract

The sycophagines are strictly associated with two subgenera of *Ficus* L. (Moraceae), namely *Sycomorus* and *Urostigma*. They mostly oviposit through the fig wall and lay their eggs within the fig flowers, being either gall-makers or parasitoids of other fig wasps. In this contribution, a new genus of Sycophaginae, *Conidarnes* Farache & Rasplus, **gen. n.**, is described with seven new species: *Conidarnes achterbergi* Farache & Rasplus, **sp. n.**; *Conidarnes bergi* Farache & Rasplus, **sp. n.**; *Conidarnes laevis* Farache & Rasplus, **sp. n.**; *Conidarnes santineloi* Farache & Rasplus, **sp. n.**; *Conidarnes subtectae* Farache & Rasplus, **sp. n.**; *Conidarnes sulcata* Farache & Rasplus, **sp. n.**; and *Conidarnes sumatranae* Farache & Rasplus, **sp. n.** Illustrations, morphological diagnoses, dichotomous keys and multi-entry online keys to species are provided. *Conidarnes* species strictly occur in the oriental region, and their distribution does not overlap with the distribution of the two other genera belonging to the same clade. Due to their relative rarity, we encourage extensive sampling of *Conosycea* figs to improve our knowledge of the genus.

## Introduction

Recent phylogenetic analyses of the Chalcidoidea have retrieved Sycophaginae as sister to the pollinating Agaoninae (Heraty et al. 2013). Consequently, the family Agaonidae is now subdivided into two subfamilies: the Agaoninae and the Sycophaginae. The Agaoninae have established a very specialized relationship with *Ficus* L. (Moraceae) (Cook and Rasplus 2003). These wasps are the main pollinators of fig trees and are capable of entering the fig inflorescences through a small pore, called the ostiole. Once inside the figs, these wasps pollinate and lay their eggs in some pistilate flowers (Galil and Eisikowitch 1968). The Sycophaginae are non-pollinating fig wasps (NPFW) that are strictly associated with two subgenera of *Ficus* (Moraceae), namely *Sycomorus* and *Urostigma*. They mostly oviposit through the fig wall and lay their eggs inside the figs within the flowers, being either gall-makers or parasitoids of other fig wasps (Bouček 1988; Cruaud et al. 2011b; Elias et al. 2008). A few species (a small species-group of Afrotropical *Sycophaga* Westwood, Cruaud et al. 2011b) enter the fig through the ostiole (Galil et al. 1970).

There are about 60 described species of Sycophaginae that occur in all tropical and subtropical regions. The subfamily Sycophaginae was retrieved as a monophyletic assemblage and divided into three main clades each of which may warrant tribal status (Cruaud et al. 2011a):

1. A first clade—sister to the remaining Sycophaginae—that only includes *Eukoebelea* Ashmead species associated with *Ficus* subsection *Malvanthera* in Australasia.
2. A clade that includes species of large and early gallmakers, belonging to three genera: i) the Australasian genus *Pseudidarnes* Girault associated with *Ficus* subsection *Malvanthera*, ii) the Neotropical *Anidarnes* Bouček associated with *Ficus* section *Americana*, and iii) a few species associated with *Ficus* section *Conosycea* that cannot be placed in any existing genus which requires the establishment of the new genus described here.
3. Sister to the previous clade, a highly diversified clade composed of the New World *Idarnes* Walker associated with *Ficus* section *Americana*, and the Old World *Sycophaga* mostly associated with *Ficus* subgenus *Sycomorus*, but also including two species associated with *Ficus* subgenus *Urostigma* section *Urostigma*.

We have recently reviewed the genera *Anidarnes* and *Pseudidarnes* with the description of nine new *Anidarnes* and six new *Pseudidarnes* species (Farache et al. 2013; Farache and Rasplus 2014). In this paper, we propose the establishment of *Conidarnes*, a new oriental genus of Sycophaginae, and describe seven new species mostly sampled from figs of the large strangling fig trees (*Conosycea*) that occur in the dipterocarp rainforests of the oriental region. We also provide illustrations, morphological diagnoses, dichotomous keys, and multi-entry online keys to species.

## Methods

Specimen handling and imaging follow Farache and Rasplus (2014). Geographical coordinates and altitudes were mostly estimated using label information. Morphological terminology follows Gibson (1997), and the HAO (Hymenoptera Anatomy Ontology) portal (Yoder et al. 2010). Species descriptions were assembled in DELTA (Dallwitz 1980). A list of characters and character states used to describe the species can be found in Suppl. material 1. Characters included in this list were matched with HAO portal codes. This may help readers to better understand the anatomical structures we used for description. The sections dealing with the material examined were prepared using AUTOMATEX (Brown 2013). Multi-entry identification keys were built using LUCID®, and are available at http://www.figweb.org.

Images were produced with a Leica M16 lens and a JVC KY-75U 3CCD digital camera. Cartograph v5.6.0 (Microvision, Evry, France) software was used for focus stacking.

Type and specimen depositories, and their respective curators are:

CBGP France, Montpellier. Centre de Biologie pour la Gestion des Populations (Emmanuelle Artige).

RMNH Netherlands, Leiden, Naturalis Biodiversity Centre (Frédérique Bakker).

RPSP Brazil, São Paulo, Ribeirão Preto, Universidade de São Paulo (Eduardo A. B. Almeida).

SAMC South Africa, Cape Town, Iziko South African Museum (Simon van Noort).

## Results

### Conidarnes

Taxon classificationAnimaliaHymenopteraAgaonidae

Farache & Rasplus gen. n.

http://zoobank.org/F3DA3DE4-65DC-4706-B53A-9694E571447F

#### Type species.

*Conidarnes santineloi* Farache & Rasplus, sp. n.

#### Diagnosis.

Antennae with 13–14 antennomeres (one or two anelli), including a stub or nipple-like terminal flagellomere. Funicular segments slightly longer than wide to transverse. Antennae inserted at the middle line of compound eyes or below. Toruli contiguous. Clypeal margin bilobed. Malar sulcus absent. Petiole very short, transverse. Ovipositor sheaths without a median constriction and depigmentation.

#### Generic description.

*Females. Size and colour.* Body length 1.5–4.0 mm. Length of the ovipositor sheaths 0.4–6.4 mm. Body colour variable. Antennae mostly yellow, sometimes with orange or brown tinges. Head and mesosoma brown to black, usually with green, blue and orange metallic lustre. Legs yellow to brown. Coxae sometimes concolorous with mesosoma. Wings hyaline, sometimes medially infuscate in males. Metasoma usually brown black, sometimes yellow.

*Head.* Antenna with 13 or 14 antennomeres (including a stub or nipple-like terminal antennomere), usually with two anelli but sometimes with a single anellus (antennal formula 11263 or 11163). Terminal antennomere (*i.e.* a nipple-like thirteenth or fourteenth antennomere) sometimes conspicuous. Funicular segments slightly longer than wide to transverse. Face sculpture usually reticulate, sometimes slightly engraved. Upper face sometimes smooth. Antennae inserted at the middle line of compound eyes or below. Toruli contiguous, distance between toruli always smaller than one torulus diameter. Clypeal margin bilobed. Malar sulcus absent.

*Mesosoma.* Pronotum and mesonotum sculpture variable. Pronotum longer than high in lateral view. Notauli usually complete, but incomplete in *Conidarnes laevis* sp. n. Mesoscutellar-axillar complex with straight or incurved axillular grooves and transverse frenal sulcus, forming a square mesoscutellum (an apomorphy of Sycophaginae). Mesoscutellum trapezoidal, wider near frenal sulcus and narrower near transscutal articulation. Propodeum transverse. Wings with short and sparse pilosity. Postmarginal vein inconspicuous, stub-like. Marginal vein at least as long as stigmal vein.

*Metasoma*. Petiolate, petiole very short, transverse. Margin of eighth gastral tergite deeply sinuate, A-like, with thumbnail-like medial flap (epipygium) and with a peg-like cercus arising from the membrane on either side of the epipygium (apomorphy of Sycophaginae). Length of the ovipositor sheaths varying from 0.3× (about as long as the hind tibia) to nearly twice as long as body. Ovipositor sheaths without a median constriction and depigmentation.

*Males.* Similar to females but usually slender and shorter. Exhibiting different coloration, the mesosoma sometimes mostly yellow. Wings sometimes medially infuscate.

#### Etymology.

The generic name is masculine and derived from *Idarnes* Walker, 1843, in the same manner than other sycophagine genera (*Pseudidarnes* Girault, 1927 and *Anidarnes* Bouček, 1993) and is associated to the prefix *Con* used for *Conosycea*, the host plant section of the included species. The origins of the name *Idarnes* were discussed in Farache et al. (2013).

#### Key to species

**Table:** 

1	Notauli incomplete (Fig. 6D). Upper face smooth and lower face reticulate (Fig. 6C). *Ficus kerkhoveni*	***laevis* sp. n.**
–	Notauli complete. Face entirely reticulate	**2**
2	Sculpture of mesoscutum and mesoscutellum mostly smooth, lateral lobes of mesoscutum engraved reticulate (Fig. 12D). Scrobe with a median longitudinal sulcus, extending from median ocellus to interantennal area (Fig. 12C). *Ficus altissima*	***sulcata* sp. n**
–	Sculpture of mesoscutum and mesoscutellum reticulate, lateral lobes of mesoscutum reticulate. Scrobe without a median longitudinal sulcus	**3**
3	Antenna with one anellus (Fig. 7B). Funicular segments mostly transverse (Fig. 7B). *Ficus pallescens*	***santineloi* sp. n.**
–	Antenna with two anelli. Funicular segments mostly as long as wide or slightly longer than wide.	**4**
4	Ovipositor approximately 1.5× as long as body (Fig. 1A). Metasoma ventrally yellow and dorsally dark brown (Fig. 1A)	***achterbergi* sp. n.**
–	Ovipositor as long as body or shorter. Coloration of metasoma homogeneously brown	**5**
5	Ovipositor nearly as long as body (Fig. 10A). Supraclypeal area wide (Fig. 10D). *Ficus subtecta*	***subtectae* sp. n.**
–	Ovipositor nearly 0.5× as long as body (Figs 3A, 14A). Supraclypeal area narrow (Figs 3D, 14C)	**6**
6	Antennae inserted near the middle line of compound eyes (Fig. 14C). Supraclypeal area higher than clypeus (Fig. 14C). Propodeum with a reticulate median line, slightly striate, and wider near the anterior margin of the propodeum (Fig. 15D). *Ficus sumatrana*	***sumatranae* sp. n.**
–	Antennae inserted at the lower line of compound eyes (Fig. 3D). Supraclypeal area shorter than clypeus (Fig. 3D). Propodeum without a well-established median line (Fig. 4B). *Ficus involucrata*	***bergi* sp. n.**

**Figure 1.**
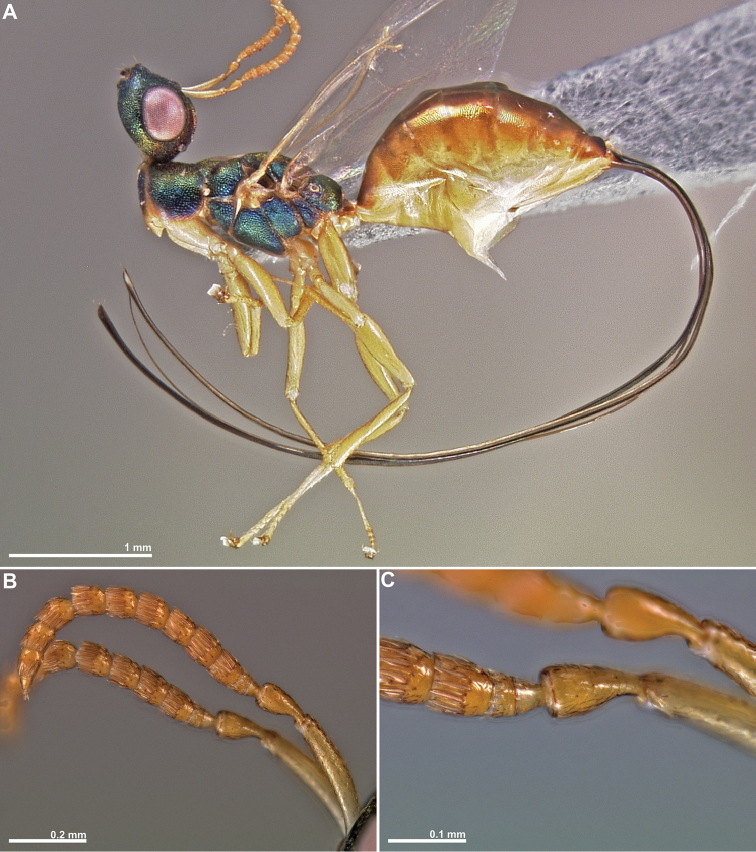
*Conidarnes achterbergi* sp. n., female. **A** *habitus* lateral view **B** antenna **C** antenna, detail.

**Figure 2.**
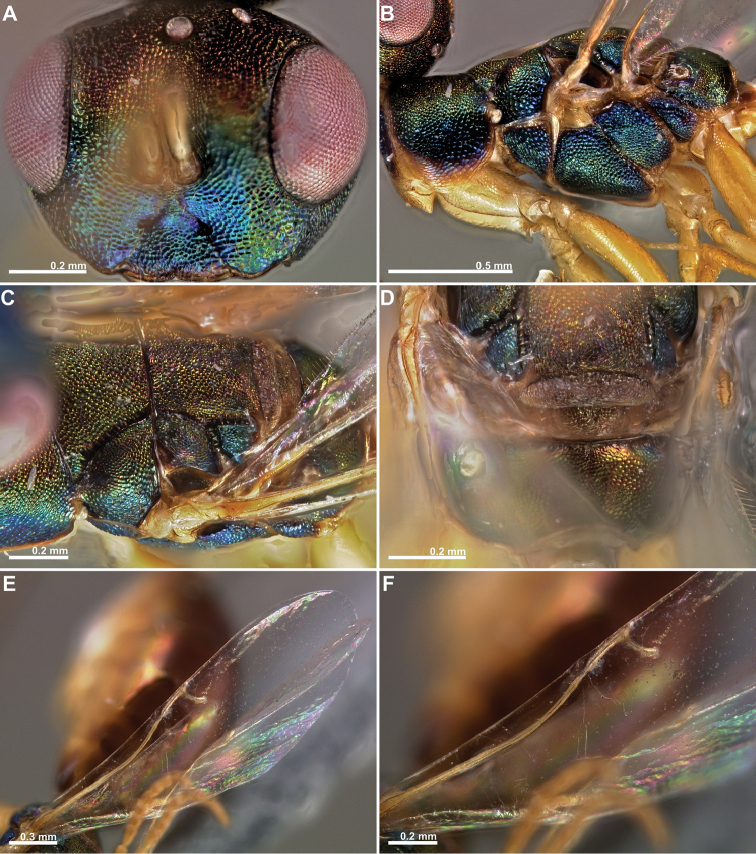
*Conidarnes achterbergi* sp. n. female. **A** head in frontal view **B** mesosoma in lateral view **C** mesosoma in dorsal view **D** propodeum and terminal mesosoma in dorsal view **E** wing **F** detail of venation.

**Figure 3.**
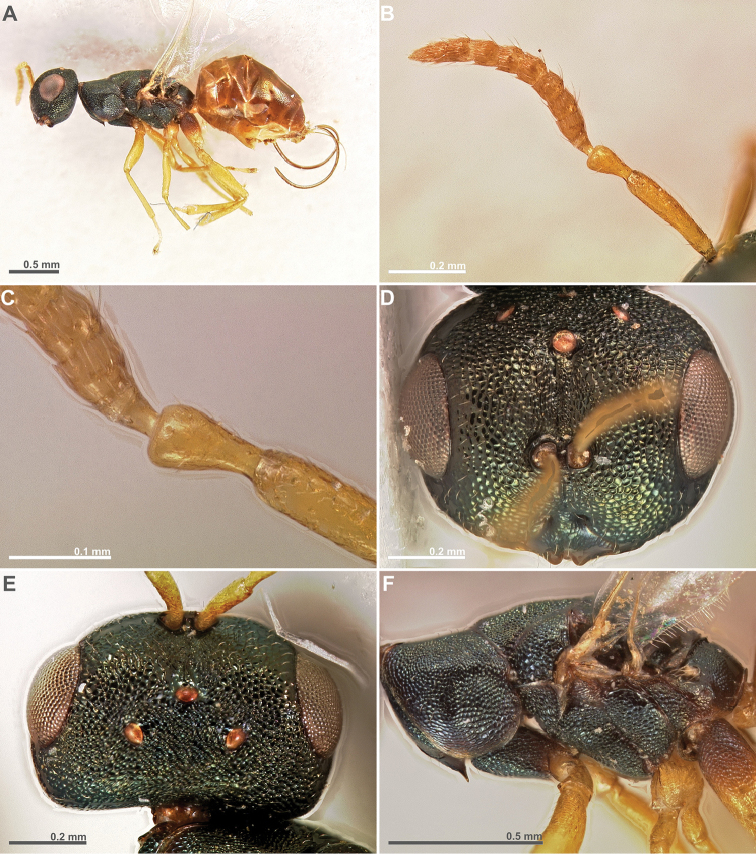
*Conidarnes bergi* sp. n. female. **A** *habitus* lateral view **B** antenna **C** antenna, detail **D** head in frontal view **E** head in dorsal view **F** mesosoma in lateral view.

**Figure 4.**
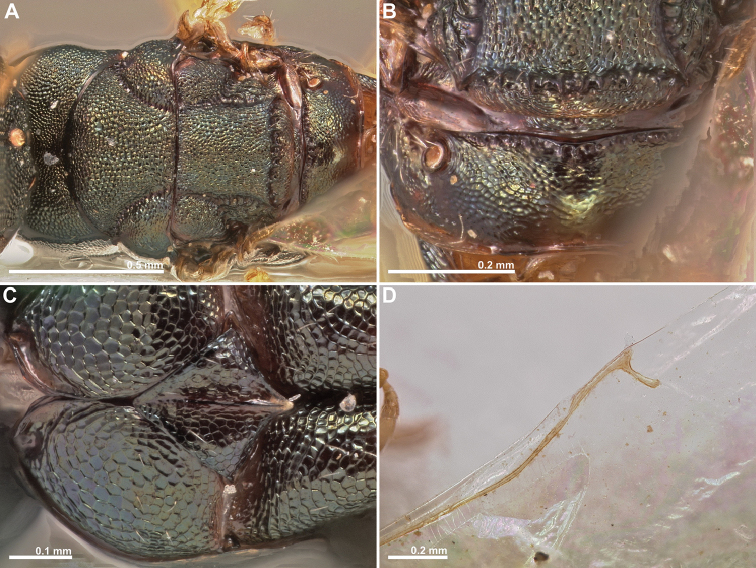
*Conidarnes bergi* sp. n. female. **A** mesosoma in dorsal view **B** propodeum and terminal mesosoma in dorsal view **C** prosternum **D** detail of venation.

**Figure 5.**
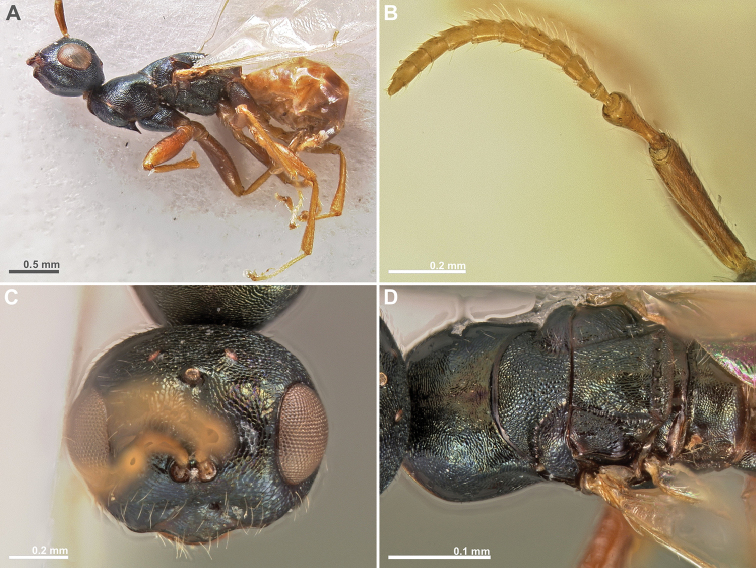
*Conidarnes bergi* sp. n. male. **A** *habitus* lateral view **B** antenna **C** head in frontal view **D** mesosoma in dorsal view.

**Figure 6.**
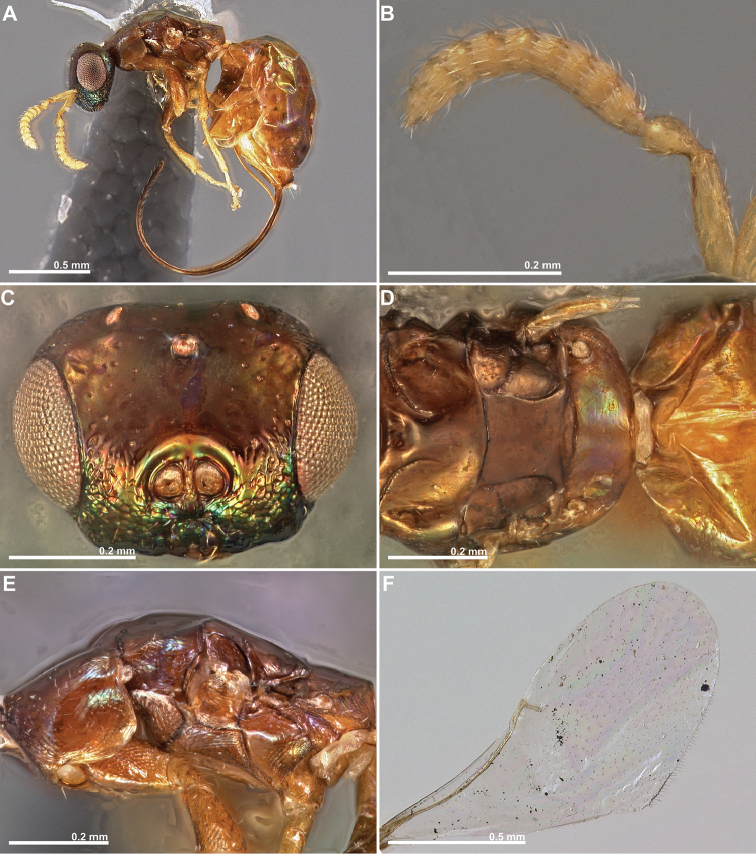
*Conidarnes laevis* sp. n. female. **A** *habitus* lateral view **B** antenna **C** head in frontal view **D** mesosoma in dorsal view (excluding pronotum) **E** mesosoma in lateral view **F** wing. Images **A, B, C, D**, and **F** by Gunther Fleck.

**Figure 7.**
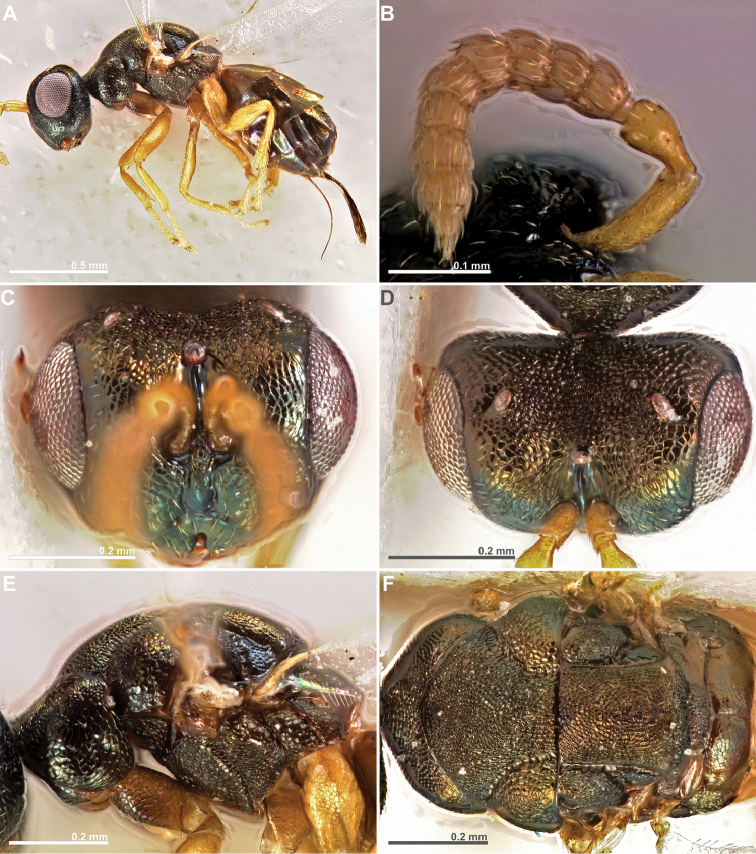
*Conidarnes santineloi* sp. n. female. **A** *habitus* lateral view **B** antenna **C** head in frontal view **D** head in dorsal view **E** mesosoma in lateral view **F** mesosoma in dorsal view.

**Figure 8.**
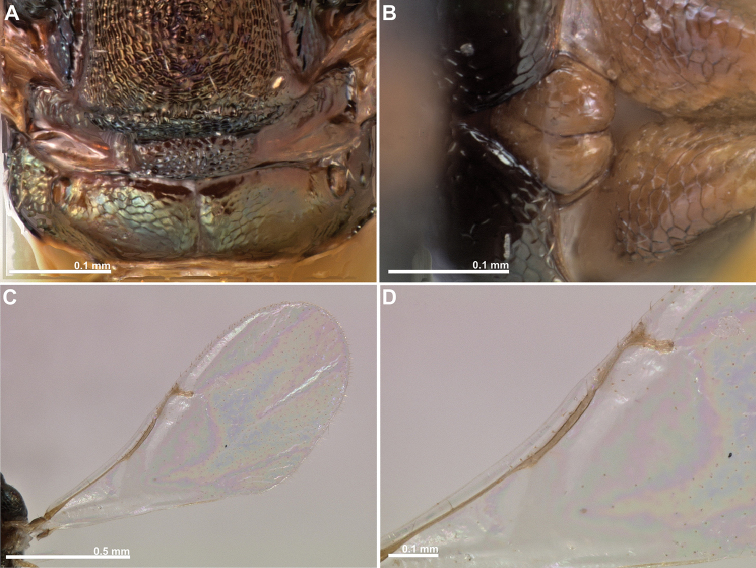
*Conidarnes santineloi* sp. n. female. **A** propodeum and terminal mesosoma in dorsal view **B** prosternum **C** wing **D** detail of venation.

**Figure 9.**
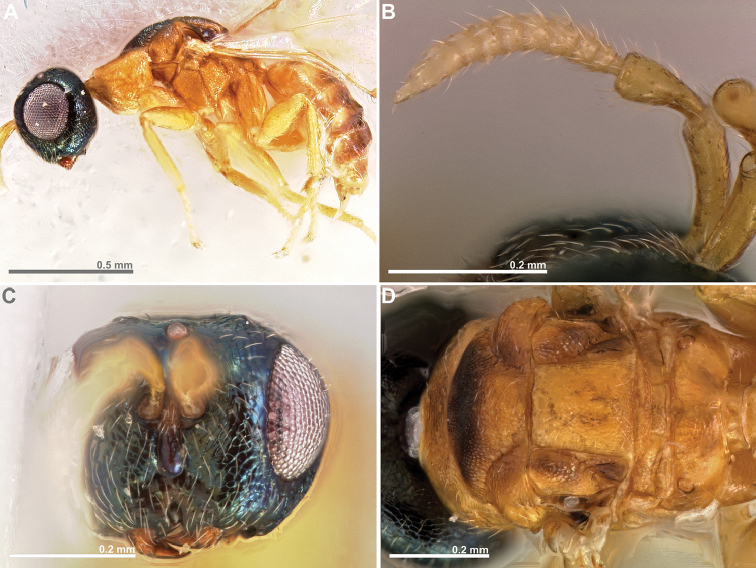
*Conidarnes santineloi* sp. n. male. **A** *habitus* lateral view **B** antenna **C** head in frontal view **D** mesosoma in dorsal view.

**Figure 10.**
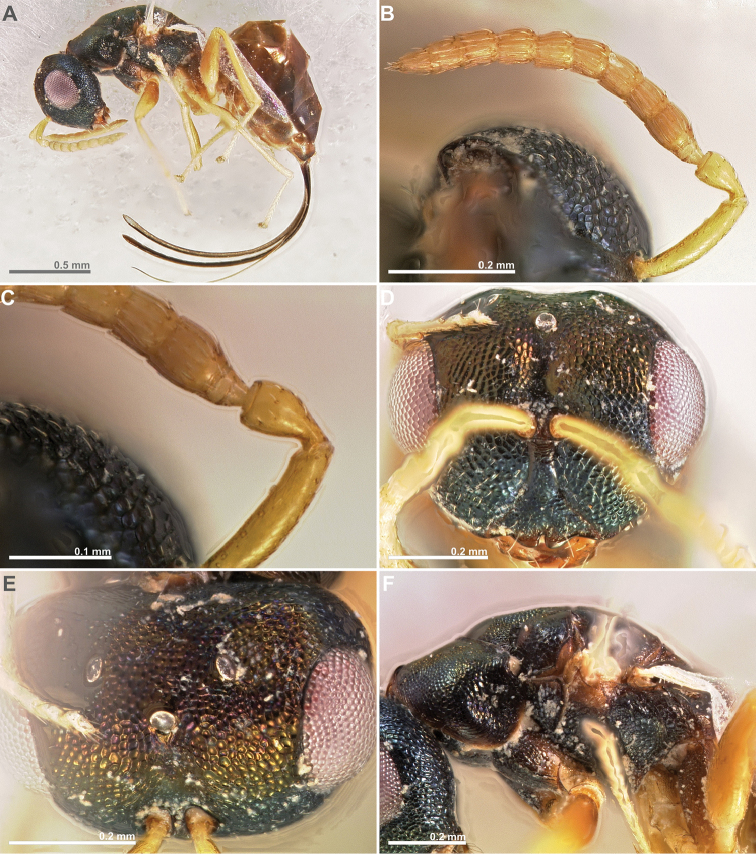
*Conidarnes subtectae* sp. n. female. **A** *habitus* lateral view **B** antenna **C** antenna, detail **D** head in frontal view **E** head in dorsal view **F** mesosoma in lateral view.

**Figure 11.**
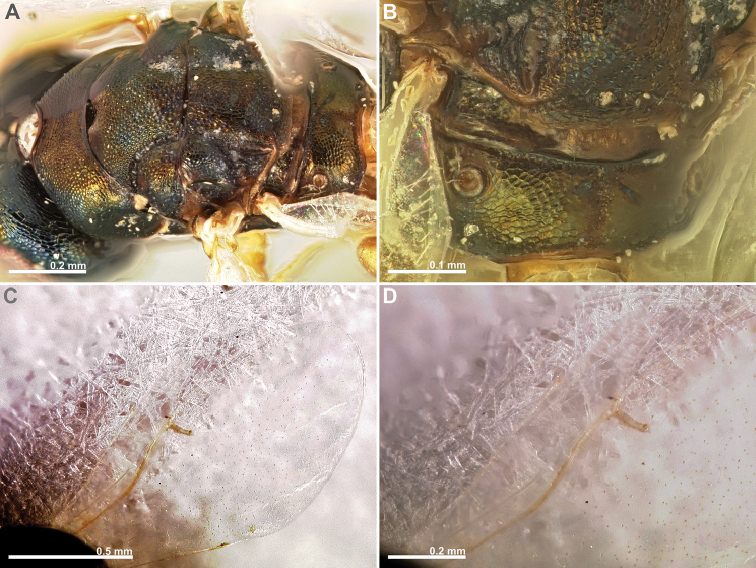
*Conidarnes subtectae* sp. n. female. **A** mesosoma in dorsal view **B** propodeum and terminal mesosoma in dorsal view **C** wing **D** detail of venation.

**Figure 12.**
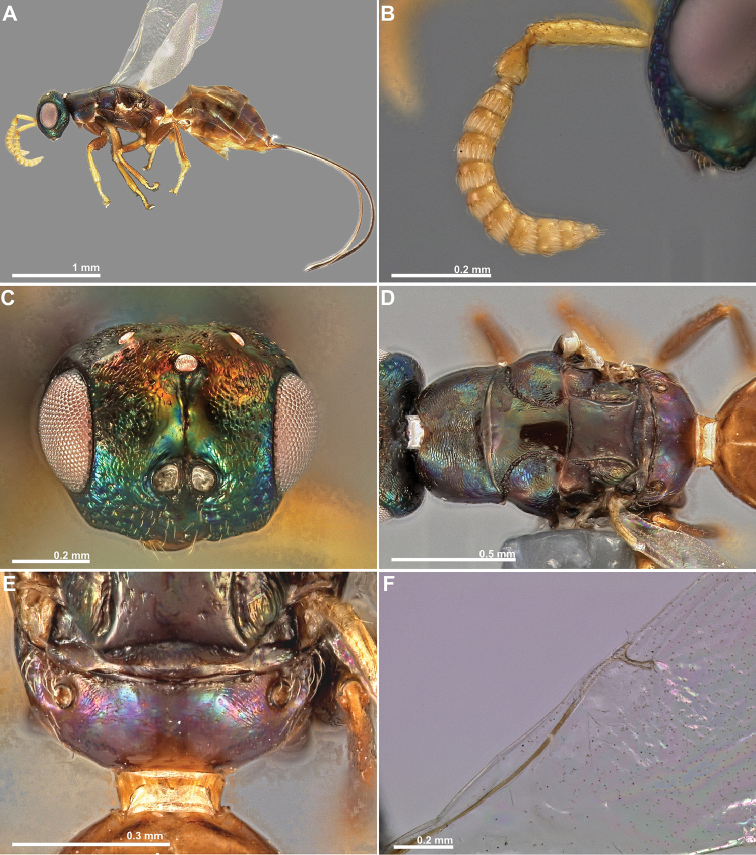
*Conidarnes sulcata* sp. n. female. **A** *habitus* lateral view **B** antenna **C** head in frontal view **D** mesosoma in dorsal view **E** propodeum and terminal mesosoma in dorsal view **F** detail of venation. Images by Gunther Fleck.

**Figure 13.**
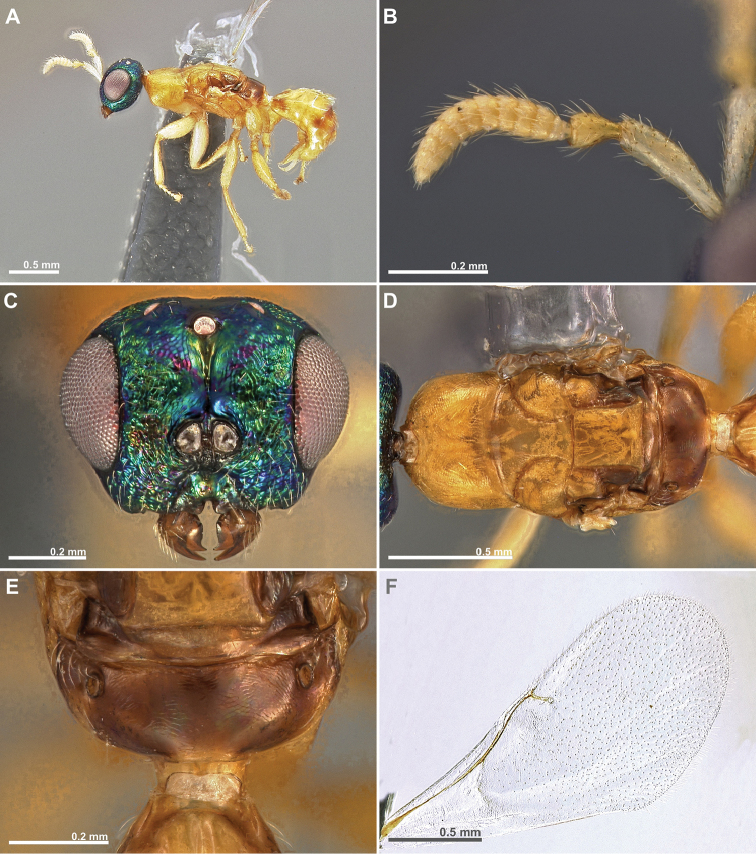
*Conidarnes sulcata* sp. n. male. **A** *habitus* lateral view **B** antenna **C** head in frontal view **D** mesosoma in dorsal view **E** propodeum and terminal mesosoma in dorsal view **F** wing. Photographs by Gunther Fleck.

**Figure 14.**
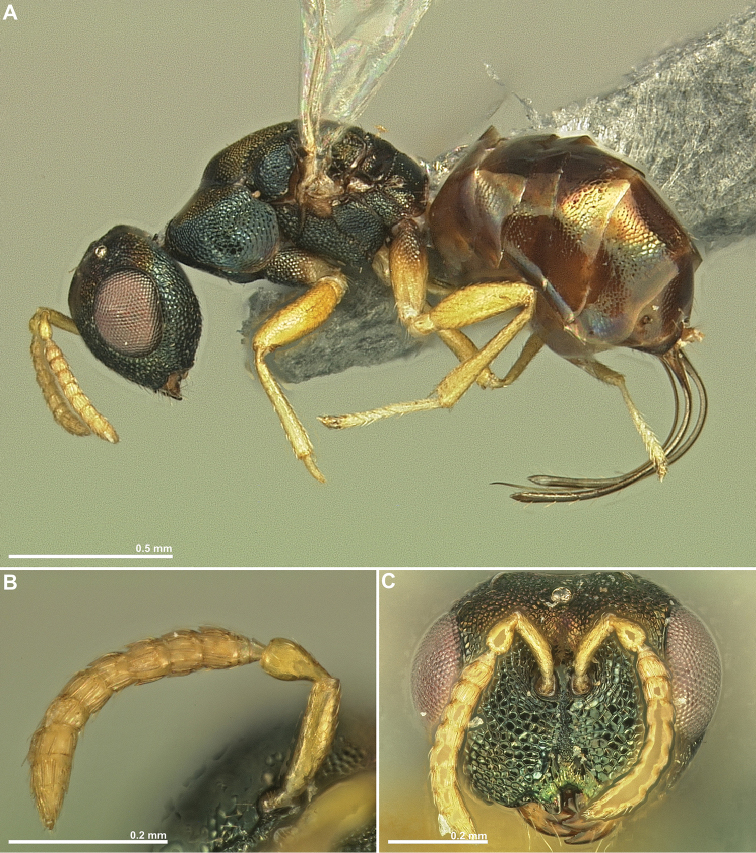
*Conidarnes sumatranae* sp. n. female. **A** *habitus* lateral view **B** antenna **C** head in frontal view.

**Figure 15.**
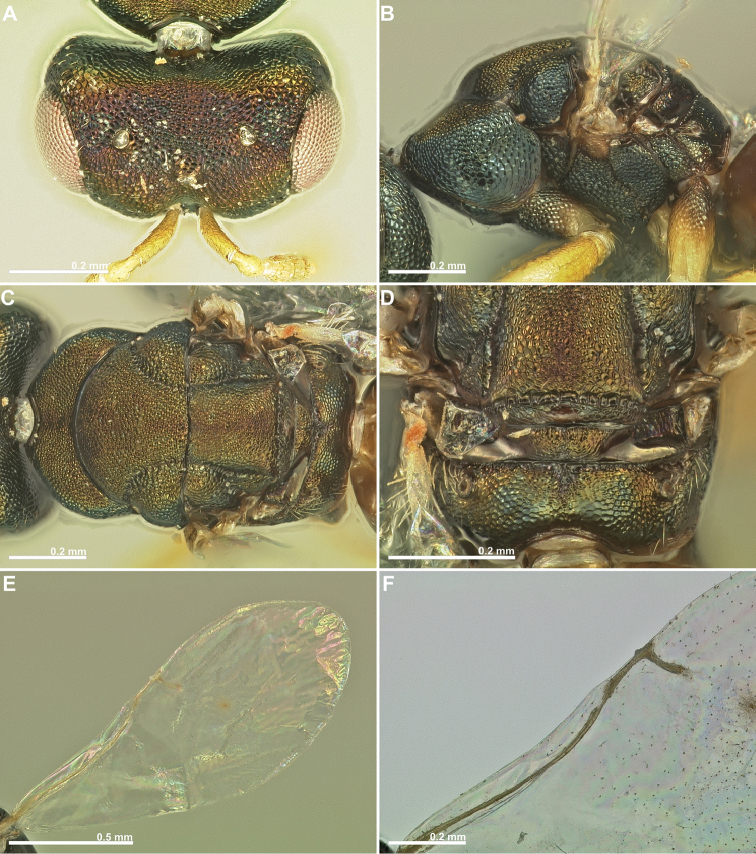
*Conidarnes sumatranae* sp. n. female. **A** head in dorsal view **B** mesosoma in lateral view **C** mesosoma in dorsal view **D** propodeum and terminal mesosoma in dorsal view **E** wing **F** detail of venation.

**Figure 16.**
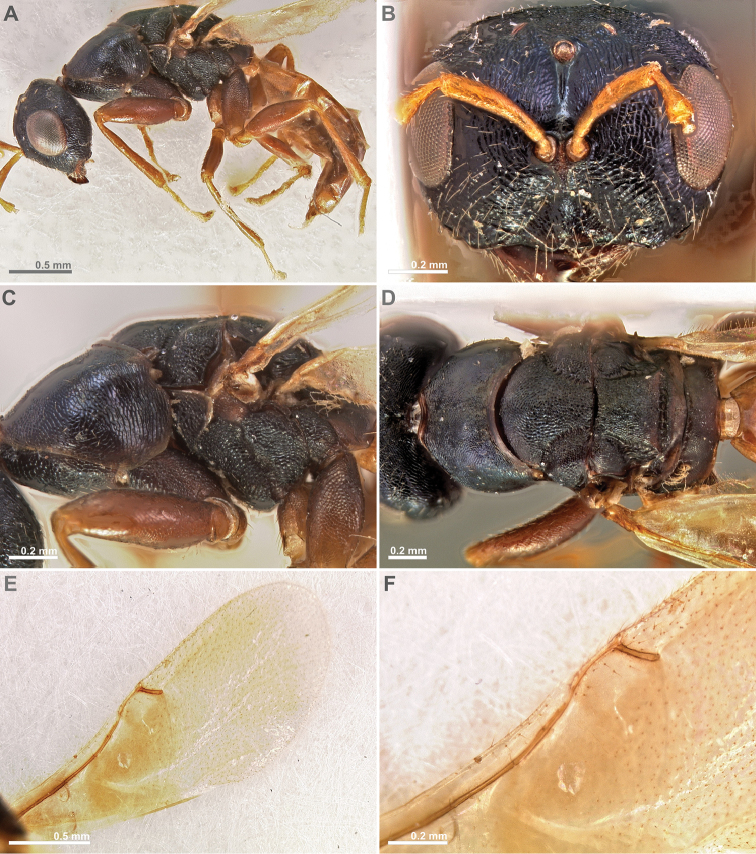
*Conidarnes* sp. ex *Ficus sundaica* male. **A** *habitus* lateral view **B** head in frontal view **C** mesosoma in lateral view **D** mesosoma in dorsal view **E** wing **F** detail of venation.

### Species descriptions

#### Conidarnes achterbergi

Taxon classificationAnimaliaHymenopteraAgaonidae

Farache & Rasplus sp. n.

http://zoobank.org/51232815-C2ED-4933-9561-0DD85A5AA95D

Figs 1 , 2

##### Holotype.

♀: **MALAYSIA: SE. Sabah**: nr. Danum Valley Field C. El. C., 4.96° 117.69°, 400m, 21–25.III.1978, v. Achterberg C, Malaise trap 7 (CBGP).

##### Diagnosis.

Metasoma ventrally yellow, dorsally dark brown. Antenna with two anelli. Antennae inserted at the lower line of compound eyes. Mesoscutum and mesoscutellum sculpture reticulate. Length of the ovipositor sheaths 1.7× body length.

##### Description.

*Female. Size and colour.* Body length 3.8 mm. Length of the ovipositor sheaths 6.4 mm. Antennae yellow orange. Head and mesosoma with metallic lustre, mostly green and blue. Head dorsally more orange. Legs yellow. Metasoma ventrally yellow, dorsally dark brown.

*Head.* Scape 4.8× as long as wide. Antenna with two anelli. Proximal anellus longer than distal anellus. Funicular segments mostly as long as wide or slightly longer than wide. Terminal antennomere conspicuous. Antennae inserted at the lower line of compound eyes. Supraclypeal area shorter than clypeus and narrow. Face sculpture reticulate. Scrobe without a median longitudinal sulcus.

*Mesosoma.* Pronotum sculpture reticulate. Pronotum elongated, nearly twice as long as high in lateral view. Mesoscutum and mesoscutellum sculpture reticulate. Notauli complete. Frenal sulcus crenulated. Metascutellum long, rectangular to trapezoidal. Anterior margin of propodeum slightly crenulated. Propodeum sculpture reticulate. Propodeum without a median line.

*Metasoma.* Length of the ovipositor sheaths 1.7× body length.

*Male.* Unknown.

##### Etymology.

The species is dedicated to our colleague and renowned specialist of Hymenoptera, Kees van Achterberg who collected the holotype.

##### Biology.

Unknown

#### Conidarnes bergi

Taxon classificationAnimaliaHymenopteraAgaonidae

Farache & Rasplus sp. n.

http://zoobank.org/847475AA-A6A3-4DE6-8210-890517AAB4BB

Figs 3 , 4 , 5

##### Holotype.

♀: **INDONESIA: Java**: Gunung Tjibodas, –6.88° 106.65°, 530m, 19.XI.1954, van der Vecht J., ex *Ficus involucrata*, Wiebes Coll. n° 114 (RMNH).

**Paratypes.** 8♀, 8♂: **INDONESIA: Java**: Gunung Tjibodas, –6.88° 106.65°, 530m, 19.XI.1954, van der Vecht J., ex *Ficus involucrata*, Wiebes Coll. n° 114, 19.XI.1954, de Gunst JH, ex *Ficus involucrata*, Wiebes Coll. n° 116 & 5103 (5♀, 5♂ RMNH; 2♀, 2♂ CBGP; 1♀, 1♂ RPSP).

##### Diagnosis.

Antennae inserted at the lower line of compound eyes. Supraclypeal area shorter than clypeus. Supraclypeal area narrow. Mesoscutum and mesoscutellum sculpture reticulate. Prosternal posterior margin medially acute. Propodeum without a median line. Length of the ovipositor sheaths 0.46× body length.

##### Description.

*Female. Size and colour* Body length 2.8 mm. Length of the ovipositor sheaths 1.3 mm. Head and mesosoma black, slightly green. Metallic lustre faint. Antennae and legs yellow, coxae concolorous with mesosoma. Metasoma brown.

*Head.* Scape 5× as long as wide. Antenna with two anelli. Proximal anellus longer than distal anellus. Funicular segments mostly as long as wide or slightly longer than wide. Terminal antennomere inconspicuous. Antennae inserted at the lower line of compound eyes. Supraclypeal area shorter than clypeus, narrow. Face sculpture reticulate. Scrobe without a median longitudinal sulcus.

*Mesosoma.* Pronotum sculpture reticulate. Pronotum elongated, nearly twice as long as high in lateral view. Prosternal posterior margin medially acute. Mesoscutum and mesoscutellum sculpture reticulate. Notauli complete. Frenal sulcus crenulated. Metascutellum short, inconspicuous. Anterior margin of propodeum crenulated. Propodeum sculpture reticulate. Propodeum without a median line.

*Metasoma.* Length of the ovipositor sheaths 0.46× body length.

*Male.* Similar to female, except the following characters: Head and mesosoma darker. Legs browner. Pedicel and funicular segments more elongated. Antenna more setose. Pronotum more elongated.

##### Etymology.

The specific name is a tribute to our colleague and friend Kees Berg (2 July 1934–31 August 2012), for his excellent and unparalleled work on the taxonomy of fig trees.

##### Biology.

Reared from syconia of *Ficus involucrata* Blume.

#### Conidarnes laevis

Taxon classificationAnimaliaHymenopteraAgaonidae

Farache & Rasplus sp. n.

http://zoobank.org/D8A7DDA5-5D19-4BC8-8A62-C5300ECCF9A6

Fig. 6

##### Holotype.

♀: **INDONESIA: E. Kalimantan**: Kutai Nature Reserve, 0.37° 117.27°, 5m, 1978, Leighton, ex *Ficus kerkhoveni*, Wiebes Coll. n° 3950 (RMNH).

##### Diagnosis.

Head, mesosoma, and metasoma mostly brown. Upper face smooth; lower face reticulate. Mesoscutum and mesoscutellum sculpture mostly smooth. Notauli incomplete. Frenal sulcus smooth. Length of the ovipositor sheaths 1× body length.

##### Description.

*Female. Size and colour.* Body length 1.7 mm. Length of the ovipositor sheaths 1.7 mm. Antennae yellow. Head dark brown, with metallic green lustre, mostly at the lower face. Mesosoma and metasoma brown. Legs proximally brown, tibiae and tarsi yellow.

*Head.* Scape 3.5× as long as wide. Antenna with two anelli. Proximal anellus shorter than distal anellus. Funicular segments mostly transverse. Terminal antennomere inconspicuous. Antennae inserted at the lower line of compound eyes. Supraclypeal area shorter than clypeus, or inconspicuous, narrow. Upper face smooth; lower face reticulate. Scrobe without a median longitudinal sulcus.

*Mesosoma.* Pronotum sculpture mostly smooth, slightly engraved. Pronotum elongated, nearly twice as long as high in lateral view. Mesoscutum and mesoscutellum sculpture mostly smooth. Notauli incomplete. Frenal sulcus smooth. Metascutellum short, inconspicuous. Anterior margin of propodeum smooth. Propodeum sculpture smooth. Propodeum without a median line.

*Metasoma.* Length of the ovipositor sheaths 1× body length.

*Male.* Unknown.

##### Etymology.

The specific name refers to the smooth body sculpture observed in this species.

##### Biology.

Reared from syconia of *Ficus kerkhovenii* Koord. & Valeton.

##### Note.

This species presents unique characters, such as a smooth body with no sculpture and an elongated mesosoma. These characters are mostly associated to galler fig wasps that enters the syconium through the ostiole (Cruaud et al. 2011b). Consequently, we speculate that this species may be an ostiolar galler.

#### Conidarnes santineloi

Taxon classificationAnimaliaHymenopteraAgaonidae

Farache & Rasplus sp. n.

http://zoobank.org/DD49490F-74E2-4A9E-9B24-62145DCBAC52

Figs 7 , 8 , 9

##### Holotype.

♀: **BRUNEI**: Temburong National Park, 4.554° 115.157°, 100m, 25.XI.1996, Rasplus J.Y., ex *Ficus pallescens*, n° JRAS00114 (CBGP).

**Paratypes.** 9♀, 5♂: same locality and information as holotype (7♀, 3♂ CBGP; 1♀, 1♂ SAMC; 1♀, 1♂ RPSP).

##### Diagnosis.

Antenna with one anellus. Funicular segments mostly transverse. Mesoscutum and mesoscutellum sculpture reticulate. Prosternal posterior margin not medially acute. Propodeum with a depressed median line. Length of the ovipositor sheaths 0.3× body length.

##### Description.

*Female. Size and colour.* Body length 1.6 mm. Length of the ovipositor sheaths 0.45 mm. Head, mesosoma, and metasoma black, slightly green. Metallic lustre faint. Antennae and legs yellow, forecoxae brown.

*Head.* Scape 3.5× as long as wide. Antenna with one anellus. Funicular segments mostly transverse. Terminal antennomere inconspicuous. Antennae inserted just below the middle line of compound eyes. Supraclypeal area higher than clypeus, wide. Face sculpture reticulate. Scrobe without a median longitudinal sulcus.

*Mesosoma.* Pronotum sculpture reticulate. Pronotum not elongated, 1.5× as long as wide in lateral view or less. Prosternal posterior margin not medially acute. Mesoscutum and mesoscutellum sculpture reticulate. Notauli complete. Frenal sulcus crenulated. Metascutellum long, rectangular to trapezoidal. Anterior margin of propodeum smooth. Propodeum sculpture slightly reticulate to smooth. Propodeum with a depressed median line.

*Metasoma.* Length of the ovipositor sheaths 0.3× body length.

*Male.* Similar to female except the following characters: Mesosoma and metasoma yellow. Mesoscutum, axillae, axillulae, and dorsal metasoma partially brown black, with faint metallic lustre. Legs completely yellow. Pedicel and funicular segments slender. Antenna more setose. Pronotum slender.

##### Etymology.

The specific name is dedicated to our friend and colleague Rodrigo Augusto Santinelo Pereira due to his excellent and pioneering work on fig wasps and *Ficus* in Brazil.

##### Biology.

Collected from syconia of *Ficus pallescens* L., the form with small leaves (see Berg and Corner 2005).

#### Conidarnes subtectae

Taxon classificationAnimaliaHymenopteraAgaonidae

Farache & Rasplus sp. n.

urn:lsid:zoobank.org:act:1843245F-8200-48ED-A02C-0B7460D740A9

Figs 10 , 11

##### Holotype.

♀: **BRUNEI**: Temburong, Kuala Belalong, 4.538° 115.159°, 105m, 23.IV.1997, Hossaert-Mckey M., ex *Ficus subtecta*, n° JRAS00117 (CBGP).

##### Diagnosis.

Antenna with two anelli. Funicular segments mostly as long as wide or slightly longer than wide. Antennae inserted just below the middle line of compound eyes. Supraclypeal area wide. Mesoscutum and mesoscutellum reticulate. Length of the ovipositor sheaths 0.9× body length.

##### Description.

*Female. Size and colour.* Body length 1.8 mm. Length of the ovipositor sheaths 1.6 mm. Antennae yellow. Head and mesosoma black, with faint blue, green, and orange metallic lustre. Legs mostly yellow distally. Coxae almost concolorous with body. Femora yellow brown. Metasoma dark brown.

*Head.* Scape 4.8× as long as wide. Antenna with two anelli. Proximal anellus longer than distal anellus. Funicular segments mostly as long as wide or slightly longer than wide. Terminal antennomere conspicuous. Antennae inserted just below the middle line of compound eyes. Supraclypeal area higher than clypeus, and wide. Face sculpture reticulate. Scrobe without a median longitudinal sulcus.

*Mesosoma.* Pronotum sculpture reticulate. Pronotum elongated, nearly twice as long as high in lateral view. Mesoscutum and mesoscutellum sculpture reticulate. Notauli complete. Frenal sulcus crenulated. Metascutellum long, rectangular to trapezoidal. Anterior margin of propodeum crenulated. Propodeum sculpture reticulate. Propodeum with a depressed median line.

*Metasoma.* Length of the ovipositor sheaths 0.9× body length.

*Male.* Unknown.

##### Etymology.

The specific name refers to the host fig species.

##### Biology.

Reared from syconia of *Ficus subtecta* Corner

#### Conidarnes sulcata

Taxon classificationAnimaliaHymenopteraAgaonidae

Farache & Rasplus sp. n.

http://zoobank.org/4584C240-8A1A-4722-9BC7-8B18DDFC6833

Figs 12 , 13

##### Holotype.

♀: **CHINA: Yunnan**: Luxi county, Mang Hai town, 24.53° 102.54°, 1750m, 28.IV.2006, Rasplus J.Y.; Peng Y.Q. & Yang D.R., ex *Ficus altissima*, n° JRAS01616_04 (CBGP).

**Paratypes.** 7♂: **CHINA: Yunnan**: Cheng Zhi village, 21.92° 101.24°, 540m, 6♂, 9.IV.2002, Gu H.Y. & Rasplus J.Y., ex *Ficus altissima*, n° JRAS0875 (5 ♂ CBGP; 1♂ SAMC); **Yunnan**: Luxi county, Mang Hai town 24.53° 102.54°, 1750m, 1♂, 28.IV.2006, Rasplus J.Y.; Peng Y.Q. & Yang D.R., ex *Ficus altissima*, n° JRAS01616_04 (CBGP).

##### Diagnosis.

Scrobe with a median longitudinal sulcus, extending from median ocellus to interantennal area. Mesoscutum and mesoscutellum sculpture mostly smooth. Lateral area of the mesoscutum mostly engraved reticulate. Propodeum sculpture smooth, slightly engraved alutaceous near spiracles. Propodeum without a median line. Length of the ovipositor sheaths 1× body length.

##### Description.

*Female. Size and colour.* Body length 2.7 mm. Length of the ovipositor sheaths 2.6 mm. Antennae yellow. Head with metallic lustre, mostly green, slightly orange and blue. Mesosoma mostly brown, with faint metallic lustre, green and blue. Legs mostly brown, tarsal segments and foretibia yellow. Metasoma dark brown.

*Head.* Scape 4.6× as long as wide. Antenna with two anelli. Proximal anellus nearly as long as distal anellus. Funicular segments mostly transverse. Terminal antennomere conspicuous. Antennae inserted at the lower line of compound eyes. Supraclypeal area inconspicuous. Face sculpture mostly reticulate, smooth near scrobe. Scrobe with a median longitudinal sulcus, extending from median ocellus to interantennal area.

*Mesosoma.* Pronotum sculpture alutaceous, engraved. Pronotum elongated, nearly twice as long as high in lateral view. Prosternal posterior margin medially acute. Mesoscutum and mesoscutellum sculpture mostly smooth. Lateral area of the mesoscutum mostly engraved reticulate. Notauli complete. Frenal sulcus smooth. Metascutellum as long as frenum, smooth, and not well delimited laterally. Anterior margin of propodeum smooth. Propodeum sculpture smooth, slightly engraved alutaceous near spiracles. Propodeum without a median line.

*Metasoma.* Length of the ovipositor sheaths 1× body length.

*Male.* Similar to female except the following characters: Mesosoma and metasoma mostly yellow. Axillulae, metanotum, and propodeum mostly brown. Some metasomal segments slightly brown dorsally. Scape and pedicel shorter, funicular segments more transverse. Antenna more setose.

##### Etymology.

The specific name refers to the longitudinal sulcus separating the scrobal cavity in this species.

##### Biology.

Reared from syconia of *Ficus altissima* Blume.

##### Comments.

*Conidarnes sulcata* was included in the phylogenetic analyses by Cruaud et al. (2011b). It was referred to as *Conidarnes* ex *Ficus altissima* (China) 1616_04w01x. The following markers are available in GenBank for this species: COI (JN001522.1), CytB (JN001596.1), EF1a (JN001659.1), and rRNA 28S (JN001493.1).

#### Conidarnes sumatranae

Taxon classificationAnimaliaHymenopteraAgaonidae

Farache & Rasplus sp. n.

http://zoobank.org/105F5966-EDA7-483F-9908-50A47BBE8A04

Figs 14 , 15

##### Holotype.

♀: **INDONESIA: Sulawesi**: Pattunuang, –5.059° 119.718°, 180m, 27.VIII.2007, Rasplus J.Y., ex *Ficus sumatrana*, n° JRAS02085_0202 (CBGP).

**Paratypes.** 1♂: same locality and information as holotype, n°JRAS02085_0201 (CBGP).

##### Diagnosis.

Antennae inserted near the middle line of compound eyes. Funicular segments mostly as long as wide or slightly longer than wide. Mesoscutum and mesoscutellum sculpture reticulate. Propodeum with a reticulate median line, slightly striate, and thicker near anterior margin. Length of the ovipositor sheaths 0.4× body length.

##### Description.

*Female. Size and colour.* Body length 1.9 mm. Length of the ovipositor sheaths 0.8 mm. Antennae yellow. Head and mesosoma black, with green and blue metallic lustre. Legs mostly yellow, forecoxae concolorous with body. Hindcoxae proximally concolorous with body. Metasoma dark brown.

*Head.* Scape 3.5× as long as wide. Antenna with two anelli. Proximal anellus longer than distal anellus. Funicular segments mostly as long as wide or slightly longer than wide. Terminal antennomere inconspicuous. Antennae inserted near the middle line of compound eyes. Supraclypeal area higher than clypeus and narrow. Face sculpture reticulate. Scrobe without a median longitudinal sulcus.

*Mesosoma.* Pronotum sculpture reticulate. Pronotum elongated, nearly twice as long as high in lateral view. Mesoscutum and mesoscutellum sculpture reticulate. Notauli complete. Frenal sulcus crenulated. Metascutellum long, rectangular to trapezoidal. Anterior margin of propodeum crenulated. Propodeum sculpture reticulate. Propodeum with a reticulate median line, slightly striate, and thicker near anterior margin.

*Metasoma.* Length of the ovipositor sheaths 0.4× body length.

*Male.* Similar to female, but slightly smaller.

##### Etymology.

The specific name refers to the host species.

##### Biology.

Reared from syconia of *Ficus sumatrana* Miq.

##### Comments.

*Conidarnes sumatranae* was included in several phylogenetic analyses (Cruaud et al. 2011a; Cruaud et al. 2011b; Farache et al. 2013) and was referred as *Conidarnes* sp. ex *Ficus sumatrana* 2085_02w01a or as Undescribed genus sp. ex. *Ficus sumatrana* (2085_02w01a). The following molecular markers are available in GenBank for this species: COI (HM770620.1), CytB (HM770576.1), EF1a (HM770522.1), and rRNA 28S (HM770682.1), they were sequenced from the male paratype that has been subsequently dried and mounted on card.

#### Conidarnes sp. ex Ficus sundaica

Taxon classificationAnimaliaHymenopteraAgaonidae

Fig. 16

##### Material examined.

5♂: **INDONESIA: E. Kalimantan**: Kutai Nature Reserve, 0.37° 117.27°, 1978, Bingham M., ex *Ficus sundaica* Bl. v. *beccariana* (King) det. Corner, Wiebes Coll. N°3543 (RMNH).

##### Description.

*Female.* Unknown.

*Male. Size and colour.* Body length 3.1 mm. Antennae yellow orange. Head and mesosoma mostly black, with metallic blue lustre. Legs mostly dark brown, proximally darker. Metasoma brown.

*Head.* Scape 5.3× as long as wide. Antennae inserted just below the middle line of compound eyes. Supraclypeal area shorter than clypeus and narrow. Face sculpture reticulate. Scrobe with a median longitudinal sulcus, extending from median ocellus to interantennal area.

*Mesosoma.* Pronotum sculpture alutaceous, engraved. Pronotum elongated, nearly twice as long as high in lateral view. Mesoscutum and mesoscutellum sculpture reticulate. Notauli complete. Frenal sulcus crenulated. Metascutellum long, rectangular to trapezoidal. Anterior margin of propodeum crenulated. Propodeum sculpture slightly reticulate to smooth. Propodeum without a median line.

##### Biology.

This species was reared from *Ficus sundaica* Blume v. *beccariana* (King).

##### Comments.

We have examined only males, but they clearly belong to an undescribed species. Since we described *Conidarnes* species mostly based on females, we prefer not to describe this species until more specimens are found.

## Discussion

In this study, we describe a new oriental genus of Sycophaginae that includes seven new species. *Conidarnes* can easily be assigned to Sycophaginae due to the presence of a square mesoscutellum and the morphology of the terminal gastral tergites/epipygium, which are synapomorphies of the subfamily (Cruaud et al. 2011b; Rasplus and Soldati 2005). The assignment of *Conidarnes* to Sycophaginae is also corroborated by previous phylogenetic analyses (Cruaud et al. 2011a; Cruaud et al. 2011b). Among the Sycophaginae, *Conidarnes* is uniquely defined by the following combination of characters: toruli contiguous; antennae inserted at, or below, the median line of compound eyes; malar sulcus absent; petiole very short, transverse.

Phylogenetically, *Conidarnes* is nested within a clade including *Pseudidarnes* and *Anidarnes*. Species belonging to this clade are large gall inducers (Cruaud et al. 2011b). This biology seems to be shared by all members of the clade, a life-history strategy that is also found in the *Idarnes incertus* species group and in a few *Sycophaga* species (Cruaud et al. 2011b). Large gall inducers oviposit early during fig development, species are overall larger and have shorter ovipositors than the other Sycophaginae species developing in the same fig (Cruaud et al. 2011b). Based on morphology and phylogenetic relationships, most *Conidarnes* species seem to be large gall-inducers, which oviposit through the syconium wall several days before pollination, though this still needs to be confirmed by behavioural observations. One peculiar species, *Conidarnes laevis*, exhibits a rather flattened and smooth body (Figs 6A, E). Such morphology may indicate that females enter the fig through the ostiole. If this biology is confirmed by field observations, it would be a second independent case of an ostiolar Sycophaginae besides species of the *Sycophaga sycomori* species group that are associated with *Ficus* subgenus *Sycomorus* in the Afrotropical region (Galil et al. 1970).

*Conidarnes* species are restricted to the Oriental region. Only one species was sampled in continental Asia (*Conidarnes sulcata*, from Xishuangbanna in southwest China), whereas all other species were sampled in the insular region of Southeast Asia: five species in Borneo (*Conidarnes achterbergi*, *Conidarnes laevis*, *Conidarnes santineloi*, *Conidarnes subtectae*, and an undescribed species ex *Ficus sundaica*), one in Java (*Conidarnes bergi*) and one in Sulawesi (*Conidarnes sumatranae*).

The distribution of *Conidarnes* does not overlap with distribution of the two other genera belonging to the same clade. Indeed, *Anidarnes* is restricted to America (Farache et al. 2013), and *Pseudidarnes* occurs in Papua New Guinea, Australia, and the Solomon Islands (Farache and Rasplus 2014). This pattern corresponds to the distribution of their host *Ficus* species: section *Americana* (host of *Anidarnes*) is Neotropical, whereas section *Malvanthera* (host of *Pseudidarnes*) mostly occurs in Australia and New Guinea. *Conidarnes* is strictly associated with *Conosycea*, a section of figs occurring from India to Solomon Islands, with two species reaching Madagascar (Berg 1989; Farache et al. 2013; Farache and Rasplus 2014). The section *Conosycea* probably originated in continental Eurasia and subsequently spread through the islands of Southeast Asia, reaching Australasia and Madagascar (Cruaud et al. 2012).

Another characteristic of Sycophaginae species belonging to the clade of large gall inducers is that they are rare and globally difficult to sample (Cruaud et al. 2011b). *Conidarnes* and *Pseudidarnes* species are among the rarest Sycophaginae (Cruaud et al. 2011b; Farache et al. 2013; Farache and Rasplus 2014). These characteristics plus the difficulty to find and sample ripening hemi-epiphytic stranglers (*Conosycea*) in the jungle explain why several species described here are only known from one or a few specimens. Sampling of *Conidarnes* is always extremely difficult and sporadic. To exemplify this point, we only sampled 6 males of *Conidarnes sulcata* despite collecting and opening about 5000 figs of *Ficus altissima* in southern China. Consequently, we only obtained sequences from a few of these species (three) and we therefore encourage extensive sampling of *Conosycea* figs to improve our knowledge of the genus.

## Supplementary Material

XML Treatment for Conidarnes

XML Treatment for Conidarnes achterbergi

XML Treatment for Conidarnes bergi

XML Treatment for Conidarnes laevis

XML Treatment for Conidarnes santineloi

XML Treatment for Conidarnes subtectae

XML Treatment for Conidarnes sulcata

XML Treatment for Conidarnes sumatranae

XML Treatment for Conidarnes sp. ex Ficus sundaica

## Supplementary materials

Supplementary material 1 Description characters and HAO codes for *Conidarnes* Data type: List of morphological characters Explanation note: List of characters and states, with HAO portal codes. http://portal.hymao.org/projects/32/public/ontology This dataset is made available under the Open Database License (http://opendatacommons.org/licenses/odbl/1.0/). The Open Database License (ODbL) is a license agreement intended to allow users to freely share, modify, and use this Dataset while maintaining this same freedom for others, provided that the original source and author(s) are credited.Fernando Henrique Antoniolli Farache, Jean-Yves Rasplus

Supplementary material 2 LUCID Key for *Conidarnes* Data type: Key to species Explanation note: Digital multi-entry key to species of *Conidarnes* in LUCID format: http://www.lucidcentral.com This dataset is made available under the Open Database License (http://opendatacommons.org/licenses/odbl/1.0/). The Open Database License (ODbL) is a license agreement intended to allow users to freely share, modify, and use this Dataset while maintaining this same freedom for others, provided that the original source and author(s) are credited.Fernando Henrique Antoniolli Farache, Jean-Yves Rasplus
