# Supplementary material for: Conidarnes, a new oriental genus of Sycophaginae (Hymenoptera, Agaonidae) associated with Ficus section Conosycea (Moraceae)
Source: Zookeys. 2015 Nov 23;(539):119–45. doi: 10.3897/zookeys.539.6529 (PMC4714059; doi:10.3897/zookeys.539.6529)
Supplement: Supplementary material 1 — Description characters and HAO codes for Conidarnes [file zookeys-539-119-s001.docx]

# Appendix 1. Illustrated DELTA character list with HAO codes

To access HAO codes pleas go to: <http://portal.hymao.org/projects/32/public/ontology/>.

Do directly access each character using the HAO code [HAO_XXXXXXX] type:

http://purl.obolibrary.org/obo/HAO_XXXXXXX

#1. Body [HAO_0000182] length/

mm/

#2. Ovipositor sheaths [HAO_0000680] length/

mm/

#3. Body colour/

#4. Scape [HAO_0000908]/

x as long as wide/

#5. Antenna [HAO_0000101] with/

1. one anellus/

2. two anelli/

#6. Proximal anellus [HAO_0000095]/

1. nearly as long as distal anellus/

2. longer than distal anellus/

3. shorter than distal anellus/

#7. Funicular segments [HAO_0001753]/

1. mostly transverse/

2. mostly as long as wide or slightly longer than wide/

#8. Terminal antennomere [HAO_0000107]/

1. conspicuous/

2. inconspicuous/

#9. Antennae inserted/

1. just below the middle line of compound eyes [HAO_0000217]/

2. at the lower line of compound eyes/

3. near the middle line of compound eyes/

#10. Supraclypeal area [HAO_0000981]/

1. higher than clypeus [HAO_0000212]/

2. shorter than clypeus/

3. inconspicuous/

#11. Supraclypeal area/

1. narrow/

2. wide/

#12. Face [HAO_0000316] sculpture [HAO_0000913]/

1. mostly reticulate, smooth near scrobe [HAO_0000912]/

2. reticulate/

3. upper face [HAO_0001044] smooth, lower face [HAO_0000502] reticulate/

4. engraved reticulate/

#13. Scrobe/

1. with a median longitudinal sulcus, extending from median ocellus [HAO_0000526] to interantennal area [HAO_0001935]/

2. without a median longitudinal sulcus/

#14. Pronotum [HAO_0000853] sculpture/

1. alutaceous, engraved/

2. reticulate/

3. mostly smooth, slightly engraved/

#15. Pronotum/

1. elongated, nearly twice as long as high in lateral view/

2. not elongated, 1.5x as long as wide in lateral view or less/

#16. Prosternal [HAO_0000873] posterior margin/

1. medially acute/

2. not medially acute/

#17. Mesoscutum [HAO_0000575] and mesoscutellum [HAO_0000574] sculpture/

1. mostly smooth/

2. reticulate/

3. mostly smooth. Lateral area of the mesoscutum [HAO_0000466] mostly engraved reticulate/

#18. Notauli [HAO_0000647]/

1. complete/

2. incomplete/

#19. Frenal sulcus [HAO_0001628]/

1. smooth/

2. crenulated/

#20. Metascutellum [HAO_0000625]/

1. short, inconspicuous/

2. long, rectangular to trapezoidal/

3. as long as frenum [HAO_0000355], smooth, and not well delimited laterally/

#21. Anterior margin of propodeum/

1. smooth/

2. crenulated/

3. slightly crenulated/

#22. Propodeum [HAO_0001249] sculpture/

1. smooth/

2. reticulate/

3. slightly reticulate to smooth/

4. smooth, slightly engraved alutaceous near spiracles [HAO_0000950]/

#23. Propodeum [HAO_0001249] /

1. with a depressed median line/

2. without a median line/

3. with a reticulate median line, slightly striate, and thicker near anterior margin/

#24. Ovipositor sheaths [HAO_0000680]/

x body length/
