# Supplementary figures and images for: Conidarnes, a new oriental genus of Sycophaginae (Hymenoptera, Agaonidae) associated with Ficus section Conosycea (Moraceae)
Source: Zookeys. 2015 Nov 23;(539):119–45. doi: 10.3897/zookeys.539.6529 (PMC4714059; doi:10.3897/zookeys.539.6529)

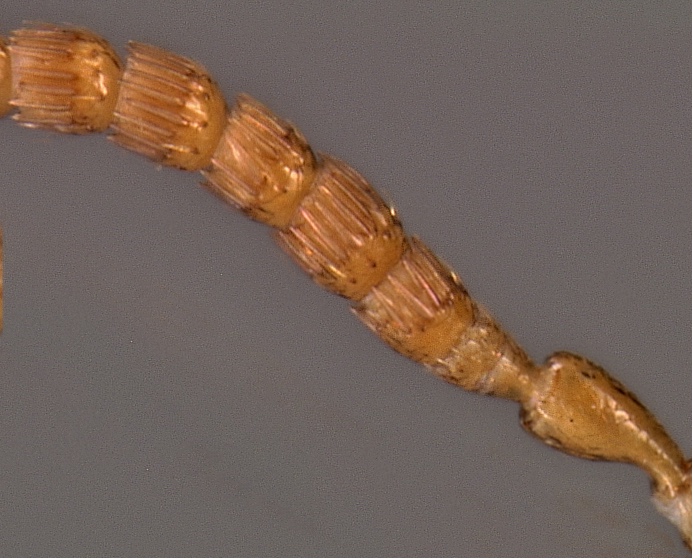

Supplement: Supplementary material 2 — LUCID Key for Conidarnes [file zookeys-539-119-s002.zip › Conidarnes_Key_lucid/Key to Species of Conidarnes Farache & Rasplus/Media/Images/01_funicular_long.jpg]

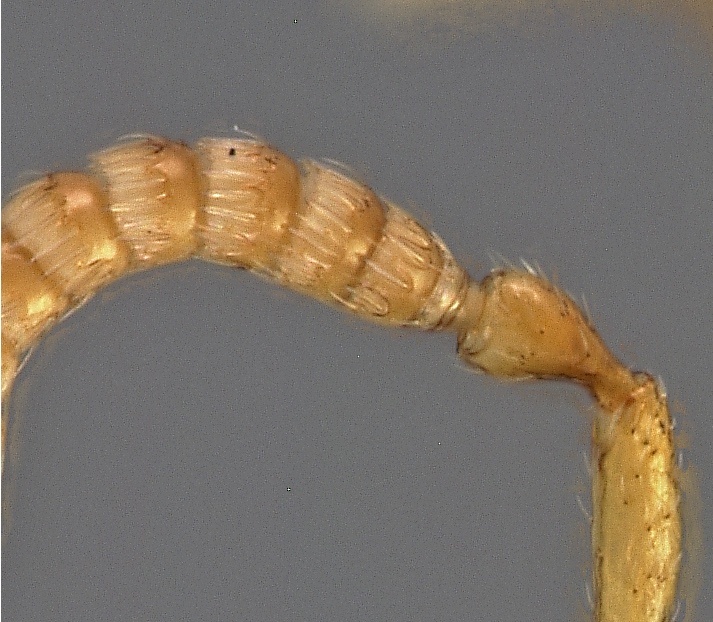

Supplement: Supplementary material 2 — LUCID Key for Conidarnes [file zookeys-539-119-s002.zip › Conidarnes_Key_lucid/Key to Species of Conidarnes Farache & Rasplus/Media/Images/01_funicular_short.jpg]

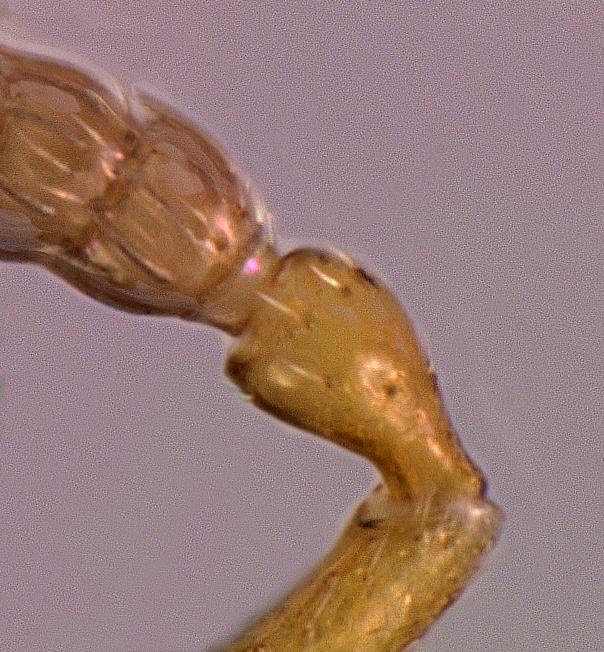

Supplement: Supplementary material 2 — LUCID Key for Conidarnes [file zookeys-539-119-s002.zip › Conidarnes_Key_lucid/Key to Species of Conidarnes Farache & Rasplus/Media/Images/02_anelli_1.jpg]

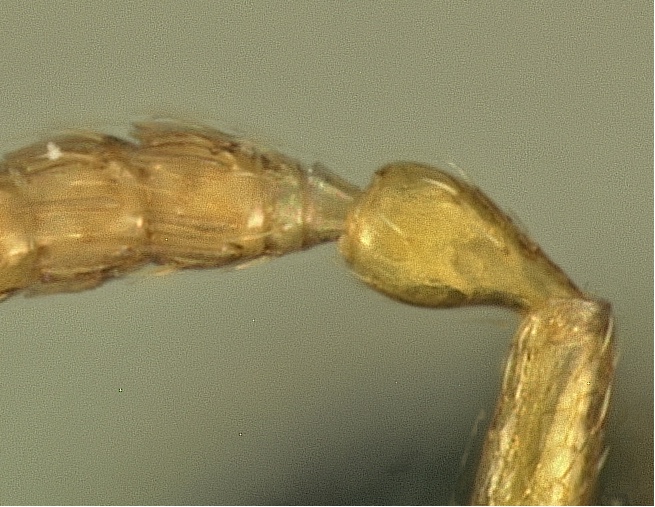

Supplement: Supplementary material 2 — LUCID Key for Conidarnes [file zookeys-539-119-s002.zip › Conidarnes_Key_lucid/Key to Species of Conidarnes Farache & Rasplus/Media/Images/02_anelli_2.jpg]

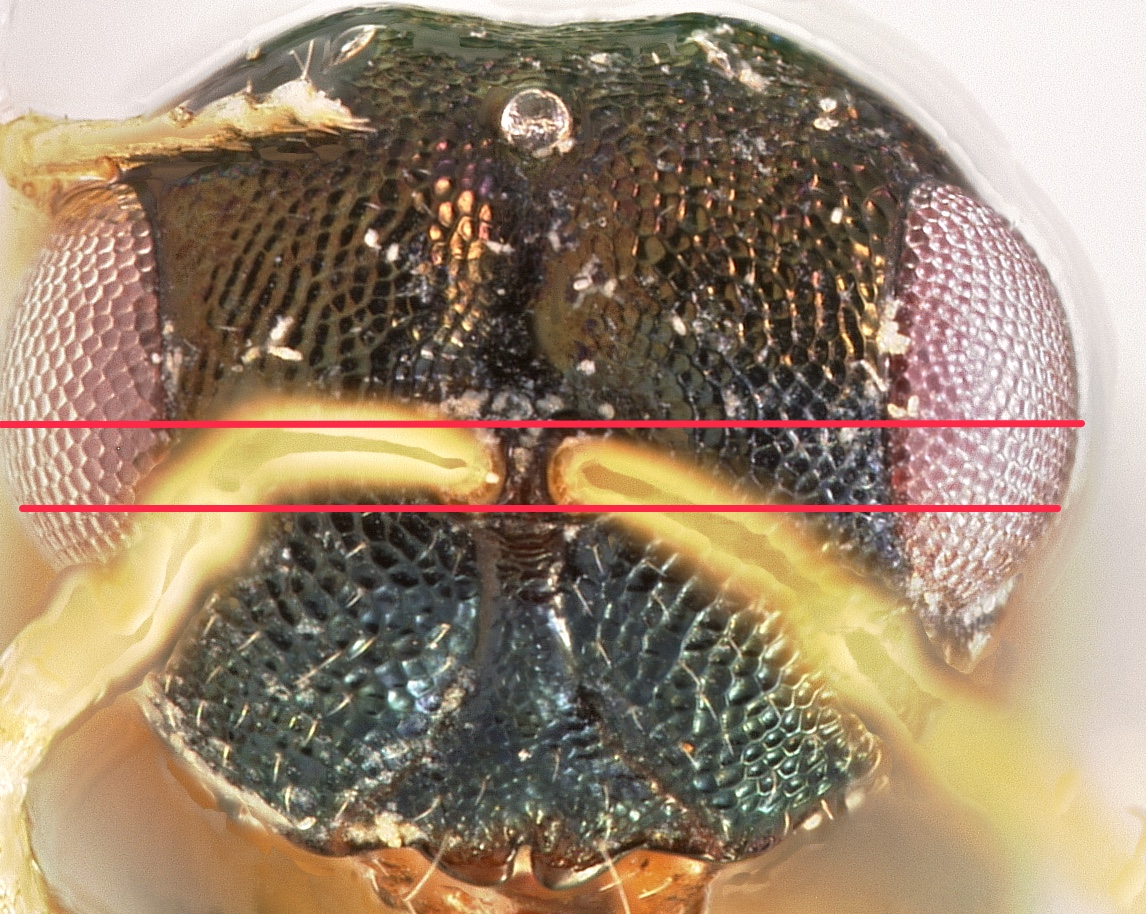

Supplement: Supplementary material 2 — LUCID Key for Conidarnes [file zookeys-539-119-s002.zip › Conidarnes_Key_lucid/Key to Species of Conidarnes Farache & Rasplus/Media/Images/03_insertion below middle.jpg]

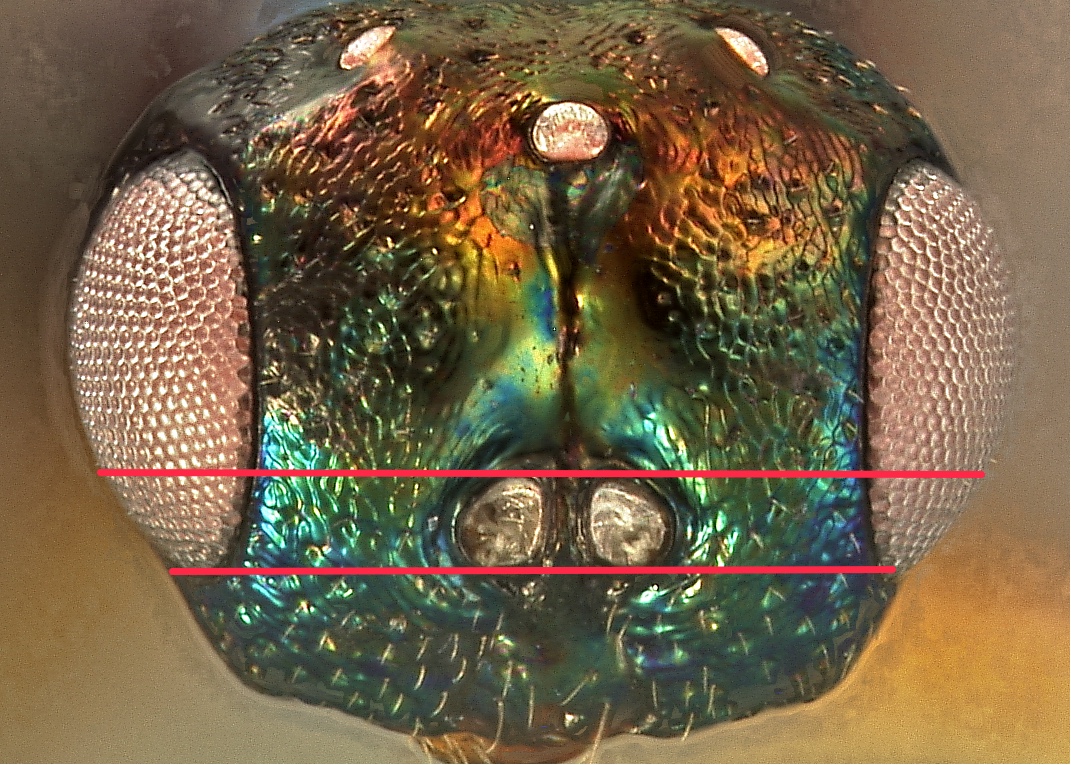

Supplement: Supplementary material 2 — LUCID Key for Conidarnes [file zookeys-539-119-s002.zip › Conidarnes_Key_lucid/Key to Species of Conidarnes Farache & Rasplus/Media/Images/03_insertion lower.jpg]

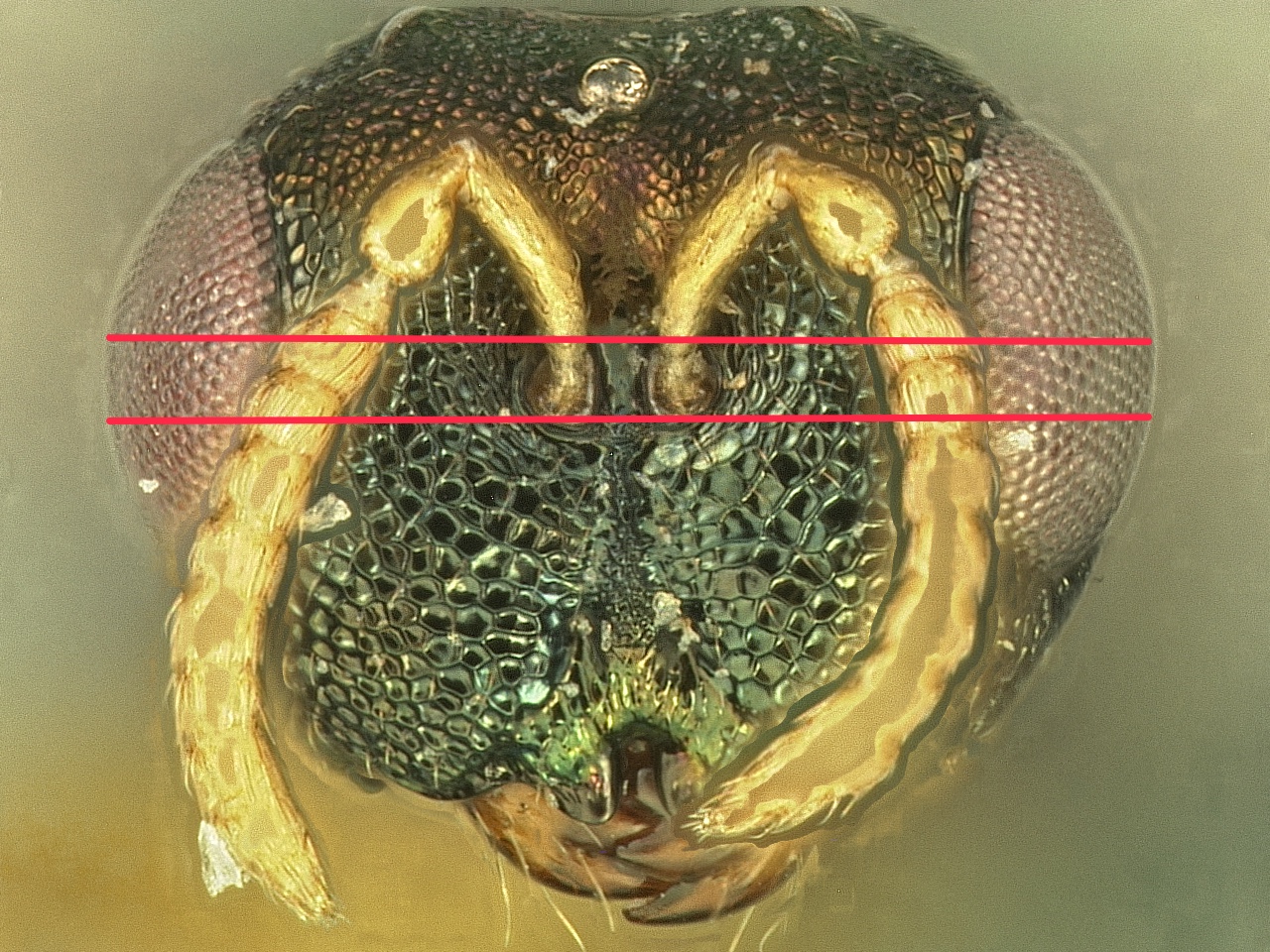

Supplement: Supplementary material 2 — LUCID Key for Conidarnes [file zookeys-539-119-s002.zip › Conidarnes_Key_lucid/Key to Species of Conidarnes Farache & Rasplus/Media/Images/03_insertion middle.jpg]

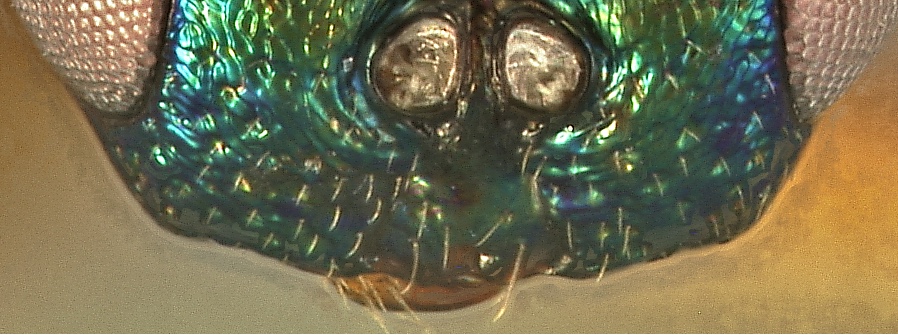

Supplement: Supplementary material 2 — LUCID Key for Conidarnes [file zookeys-539-119-s002.zip › Conidarnes_Key_lucid/Key to Species of Conidarnes Farache & Rasplus/Media/Images/04_supraclyp_inconspicuous.jpg]

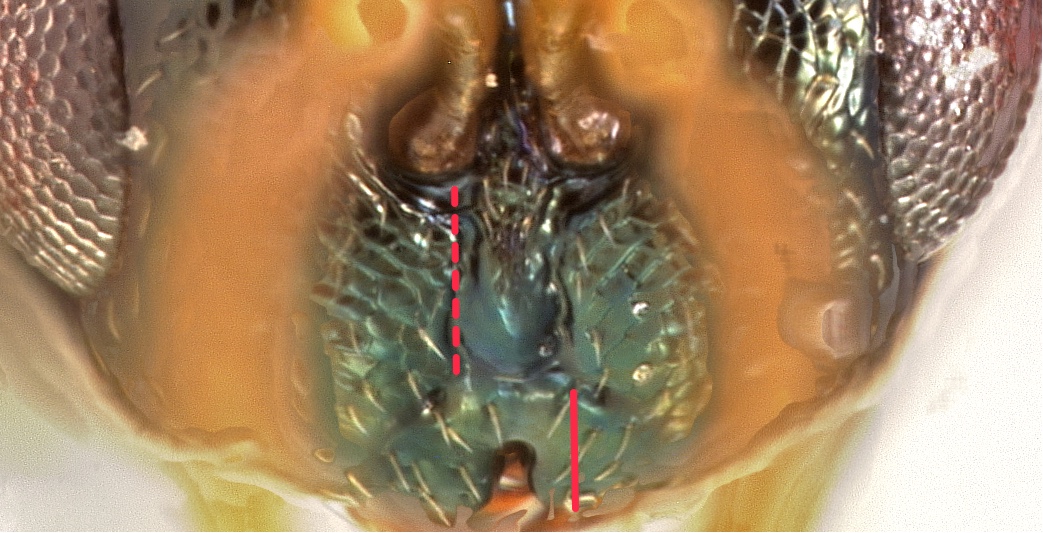

Supplement: Supplementary material 2 — LUCID Key for Conidarnes [file zookeys-539-119-s002.zip › Conidarnes_Key_lucid/Key to Species of Conidarnes Farache & Rasplus/Media/Images/04_supraclyp_longer.jpg]

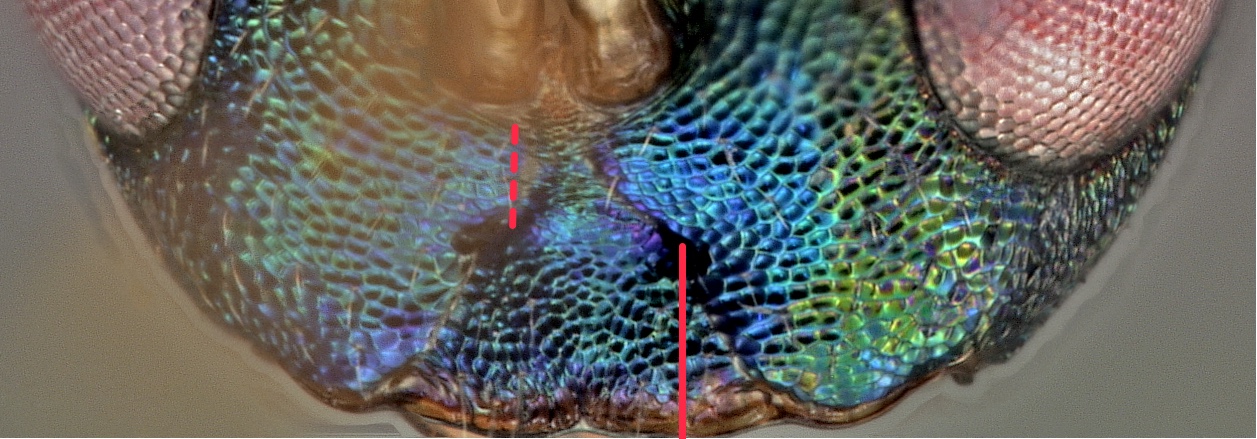

Supplement: Supplementary material 2 — LUCID Key for Conidarnes [file zookeys-539-119-s002.zip › Conidarnes_Key_lucid/Key to Species of Conidarnes Farache & Rasplus/Media/Images/04_supraclyp_shorter.jpg]

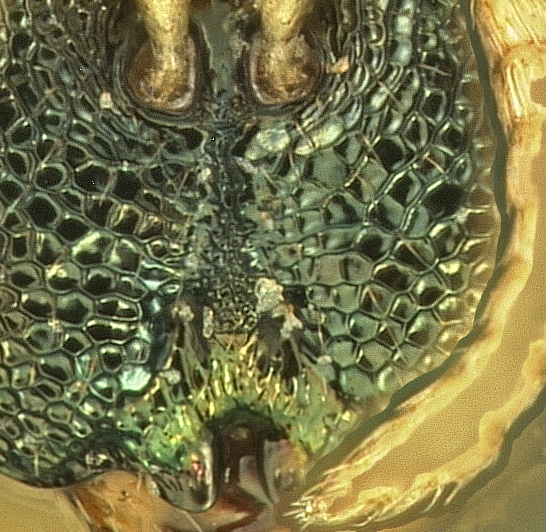

Supplement: Supplementary material 2 — LUCID Key for Conidarnes [file zookeys-539-119-s002.zip › Conidarnes_Key_lucid/Key to Species of Conidarnes Farache & Rasplus/Media/Images/05_supraclyp_narrow.jpg]

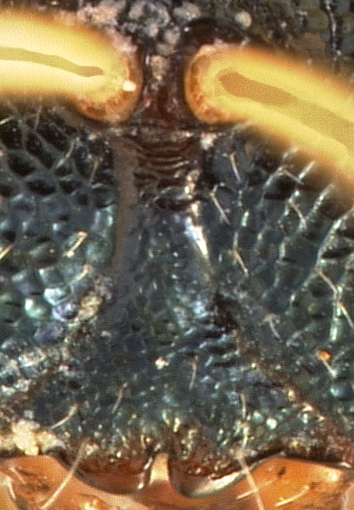

Supplement: Supplementary material 2 — LUCID Key for Conidarnes [file zookeys-539-119-s002.zip › Conidarnes_Key_lucid/Key to Species of Conidarnes Farache & Rasplus/Media/Images/05_supraclyp_wide.jpg]

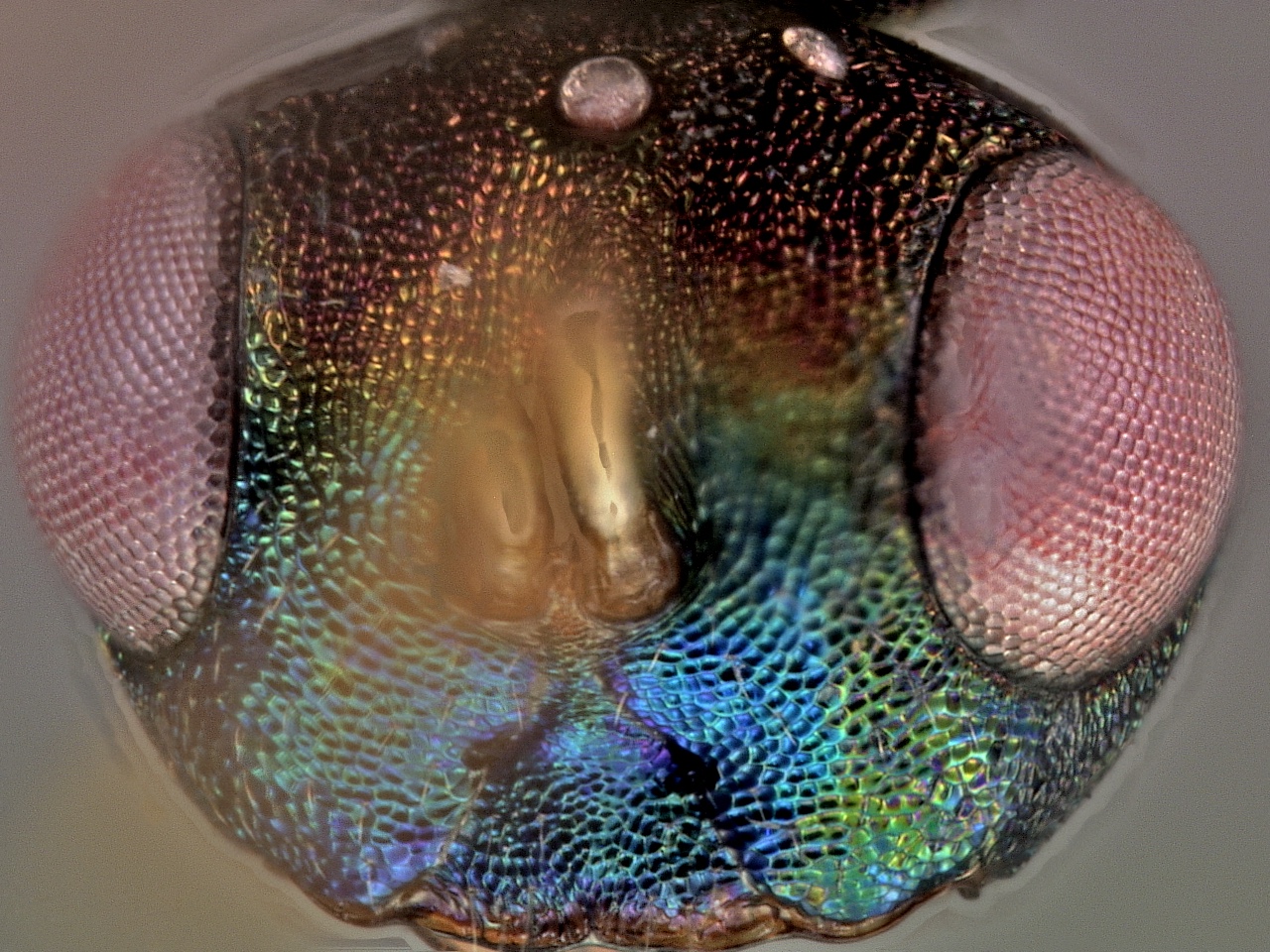

Supplement: Supplementary material 2 — LUCID Key for Conidarnes [file zookeys-539-119-s002.zip › Conidarnes_Key_lucid/Key to Species of Conidarnes Farache & Rasplus/Media/Images/06_face_reticulate.jpg]

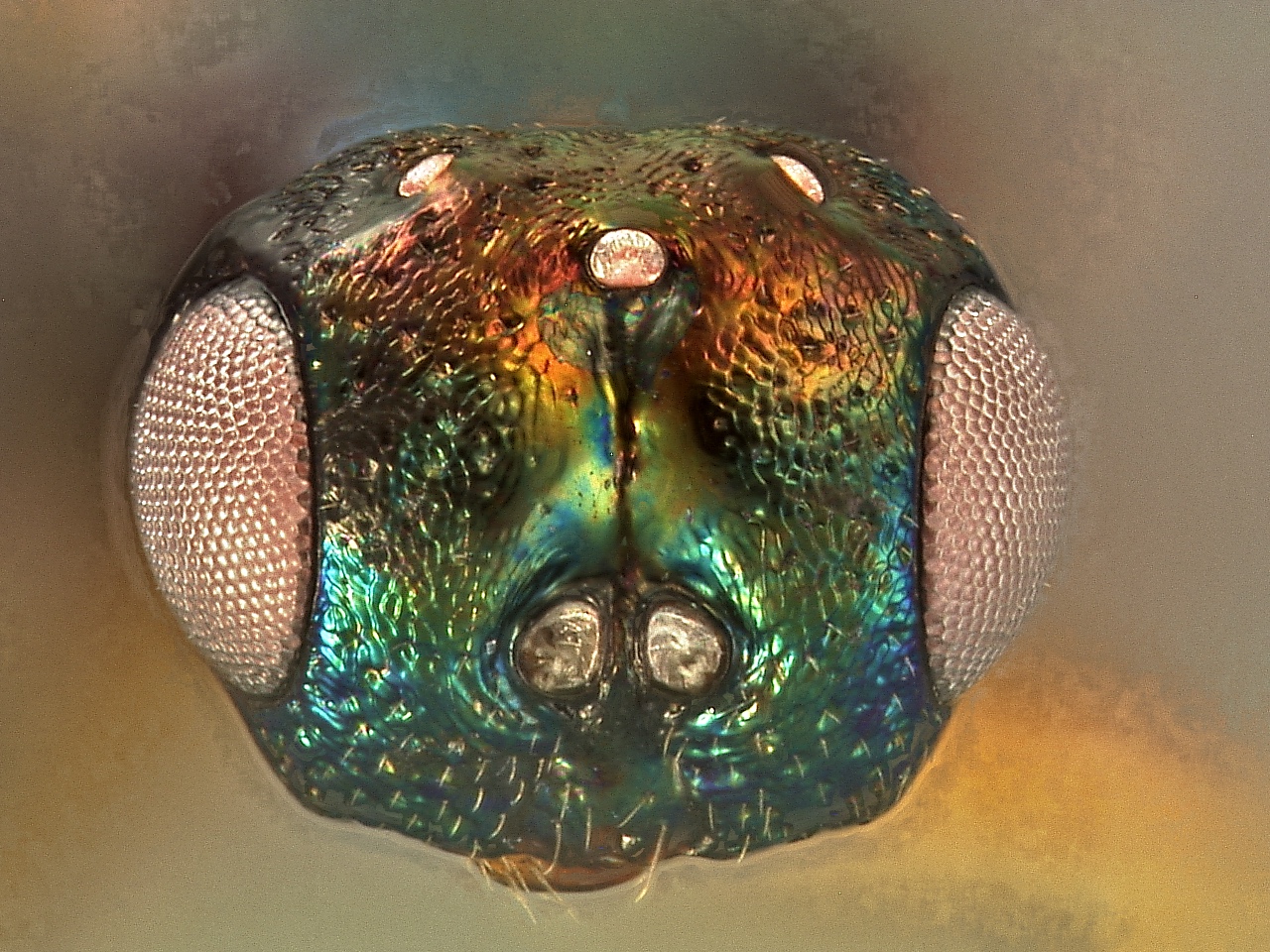

Supplement: Supplementary material 2 — LUCID Key for Conidarnes [file zookeys-539-119-s002.zip › Conidarnes_Key_lucid/Key to Species of Conidarnes Farache & Rasplus/Media/Images/06_face_reticulate_scrobe smooth.jpg]

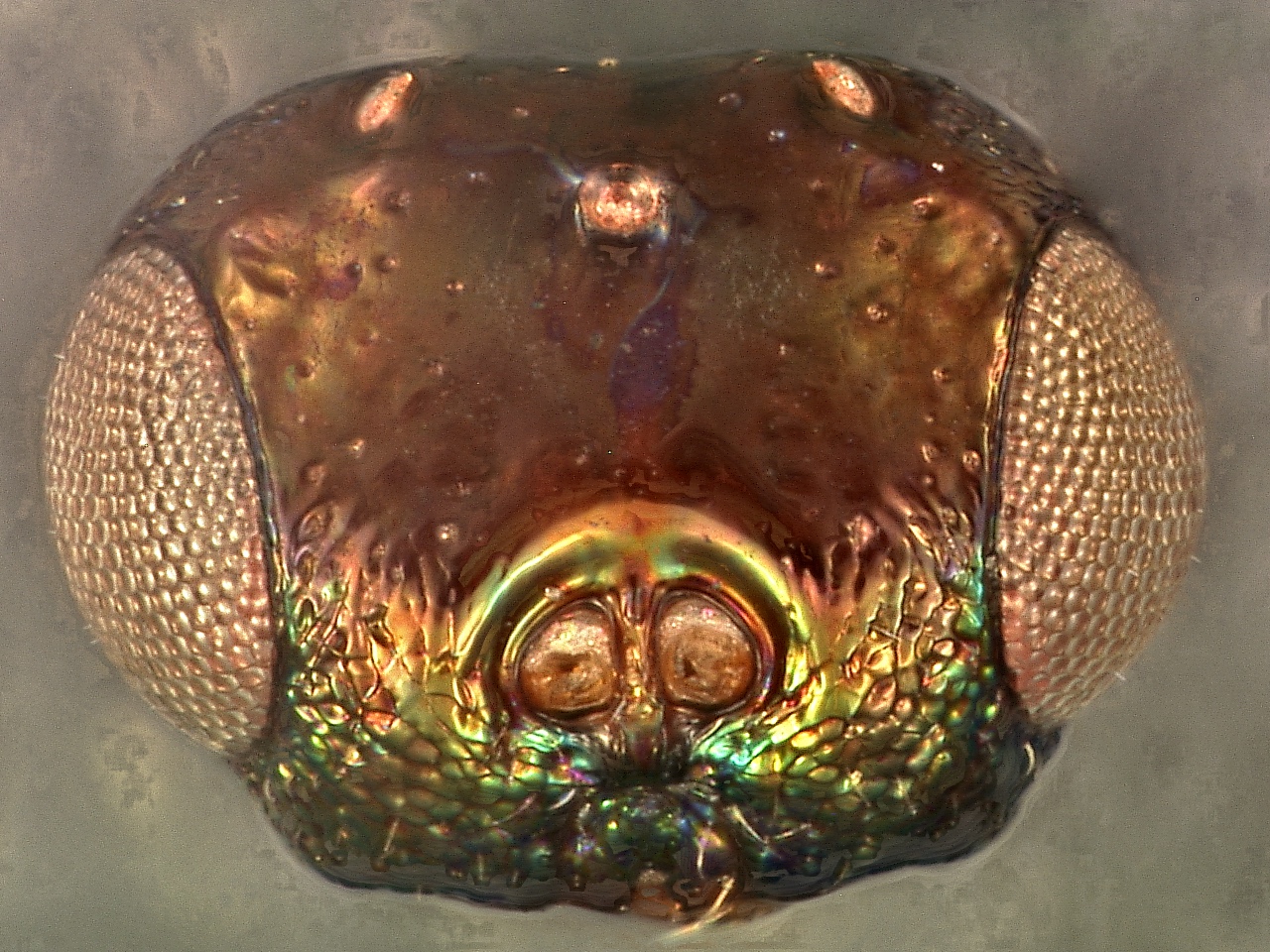

Supplement: Supplementary material 2 — LUCID Key for Conidarnes [file zookeys-539-119-s002.zip › Conidarnes_Key_lucid/Key to Species of Conidarnes Farache & Rasplus/Media/Images/06_face_upper smooth.jpg]

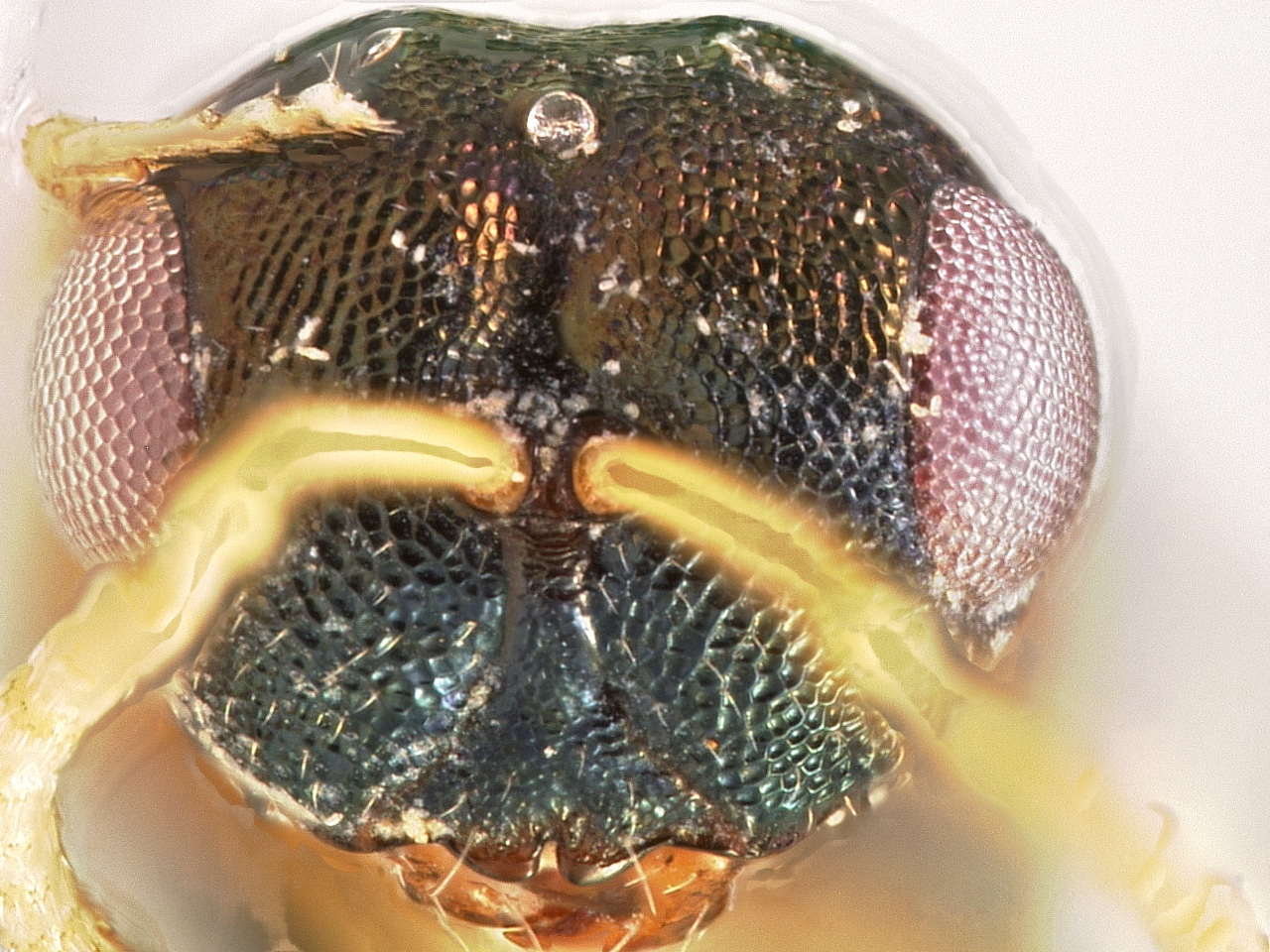

Supplement: Supplementary material 2 — LUCID Key for Conidarnes [file zookeys-539-119-s002.zip › Conidarnes_Key_lucid/Key to Species of Conidarnes Farache & Rasplus/Media/Images/07_scrobal_sulcus_absent.jpg]

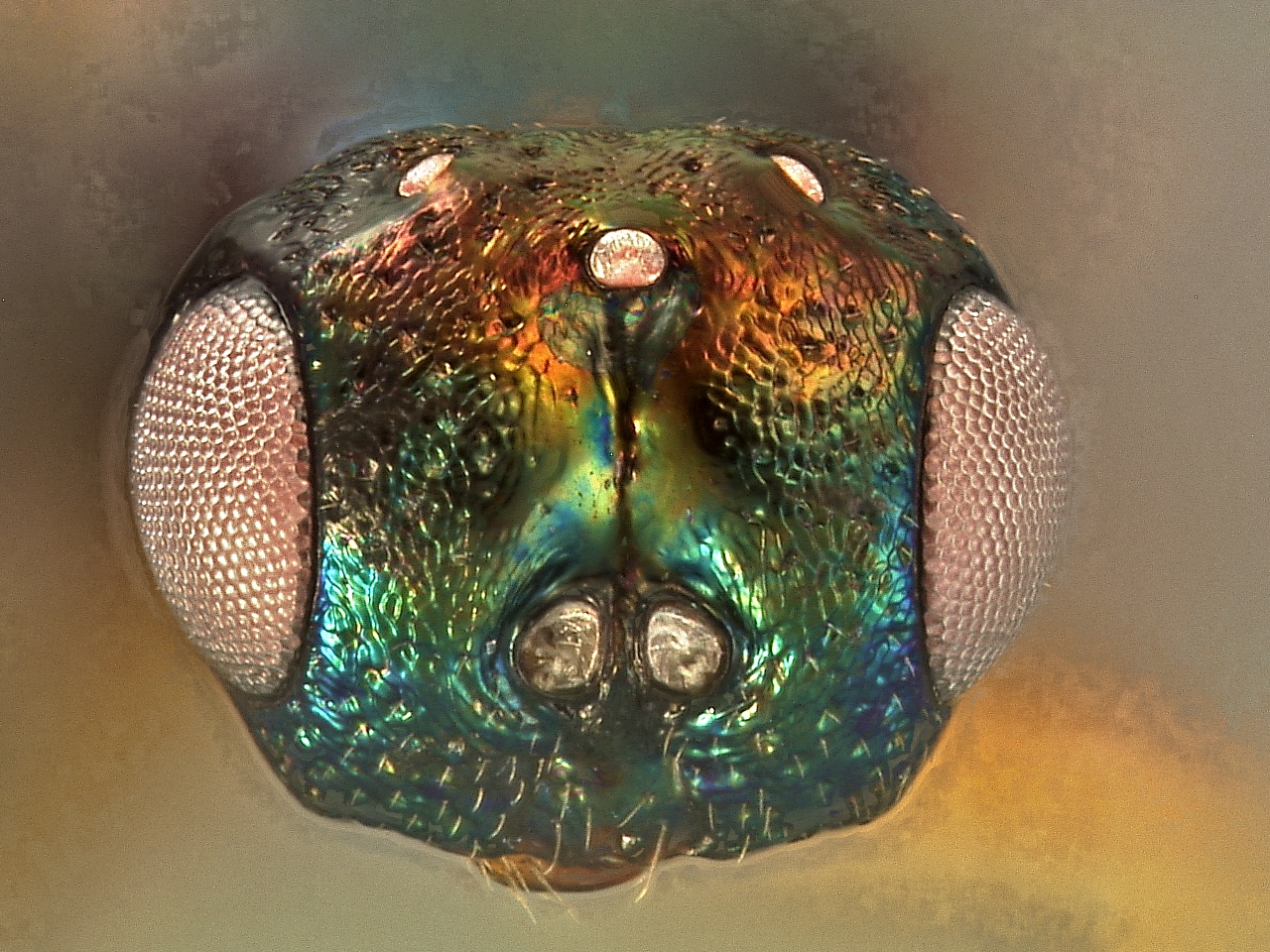

Supplement: Supplementary material 2 — LUCID Key for Conidarnes [file zookeys-539-119-s002.zip › Conidarnes_Key_lucid/Key to Species of Conidarnes Farache & Rasplus/Media/Images/07_scrobal_sulcus_present.jpg]

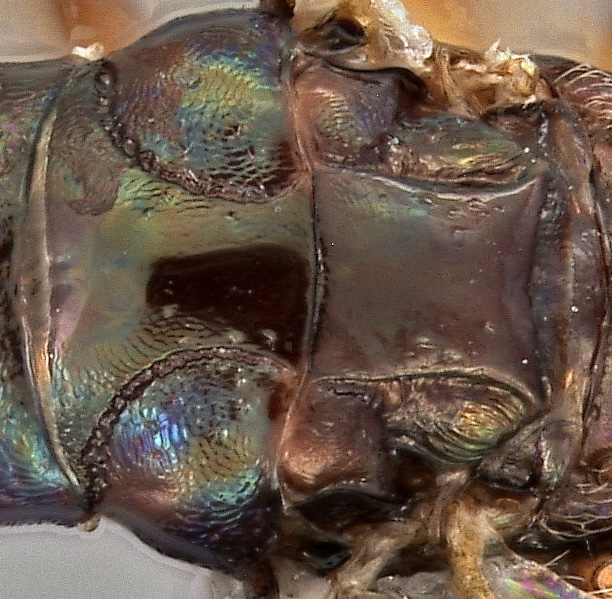

Supplement: Supplementary material 2 — LUCID Key for Conidarnes [file zookeys-539-119-s002.zip › Conidarnes_Key_lucid/Key to Species of Conidarnes Farache & Rasplus/Media/Images/08_mesoscutum_laterally engraved.jpg]

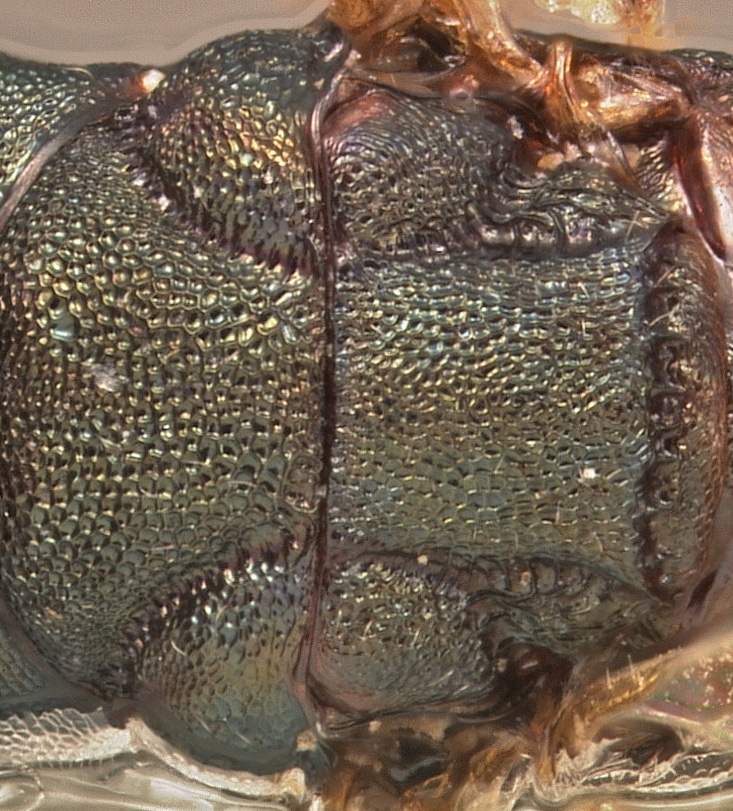

Supplement: Supplementary material 2 — LUCID Key for Conidarnes [file zookeys-539-119-s002.zip › Conidarnes_Key_lucid/Key to Species of Conidarnes Farache & Rasplus/Media/Images/08_mesoscutum_reticulate.jpg]

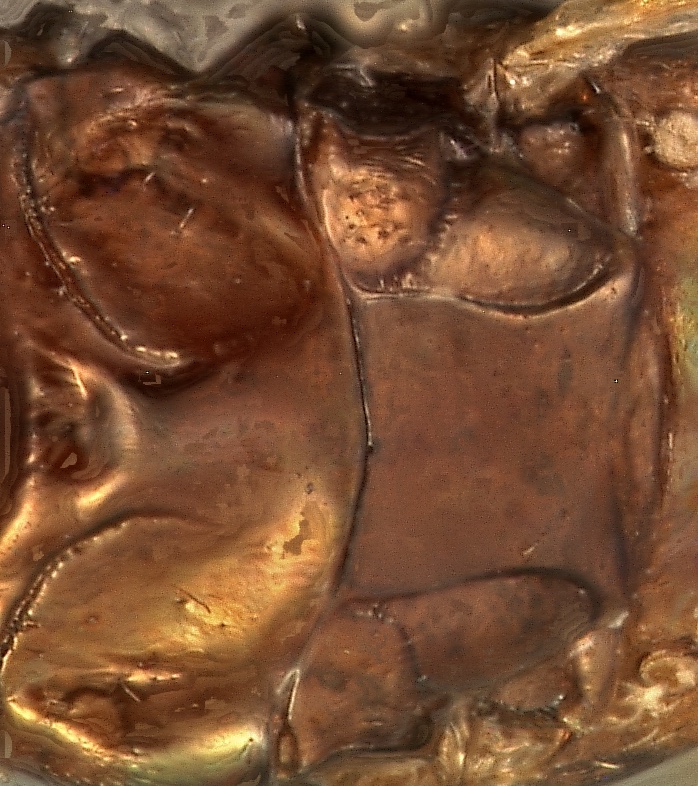

Supplement: Supplementary material 2 — LUCID Key for Conidarnes [file zookeys-539-119-s002.zip › Conidarnes_Key_lucid/Key to Species of Conidarnes Farache & Rasplus/Media/Images/08_mesoscutum_smooth.jpg]

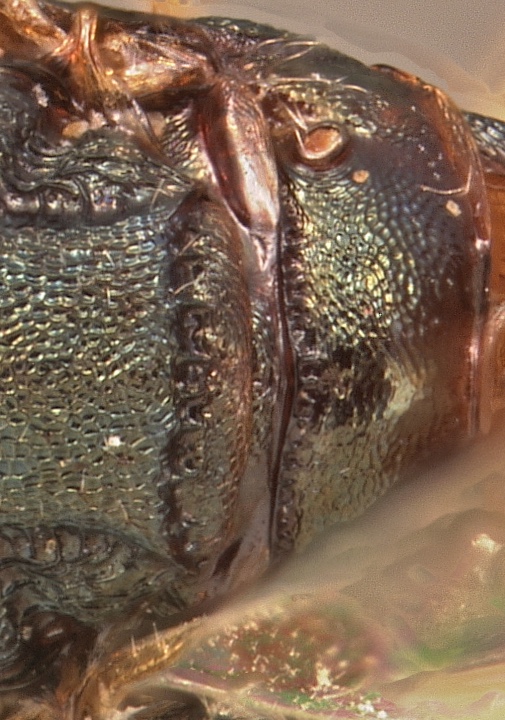

Supplement: Supplementary material 2 — LUCID Key for Conidarnes [file zookeys-539-119-s002.zip › Conidarnes_Key_lucid/Key to Species of Conidarnes Farache & Rasplus/Media/Images/10_metascutellum_inconspicuous.jpg]

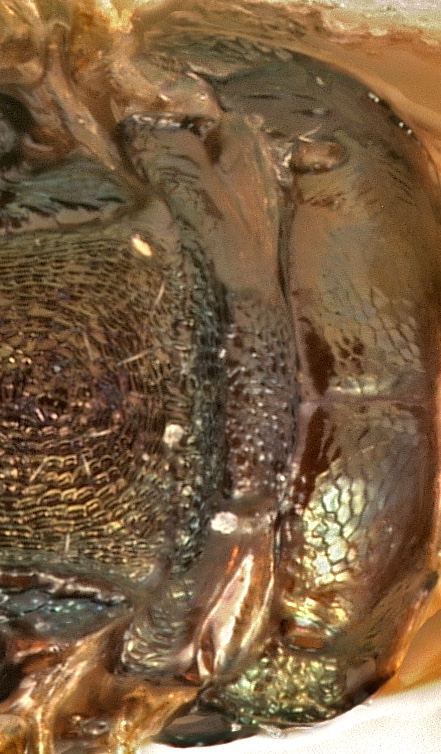

Supplement: Supplementary material 2 — LUCID Key for Conidarnes [file zookeys-539-119-s002.zip › Conidarnes_Key_lucid/Key to Species of Conidarnes Farache & Rasplus/Media/Images/10_metascutellum_long.jpg]

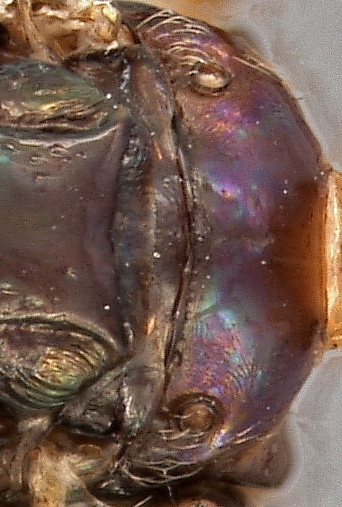

Supplement: Supplementary material 2 — LUCID Key for Conidarnes [file zookeys-539-119-s002.zip › Conidarnes_Key_lucid/Key to Species of Conidarnes Farache & Rasplus/Media/Images/10_metascutellum_smooth.jpg]

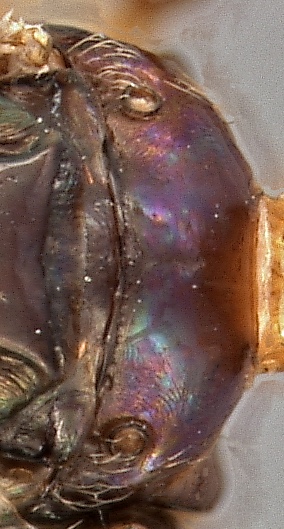

Supplement: Supplementary material 2 — LUCID Key for Conidarnes [file zookeys-539-119-s002.zip › Conidarnes_Key_lucid/Key to Species of Conidarnes Farache & Rasplus/Media/Images/11_propodeum_sulcus_absent.jpg]

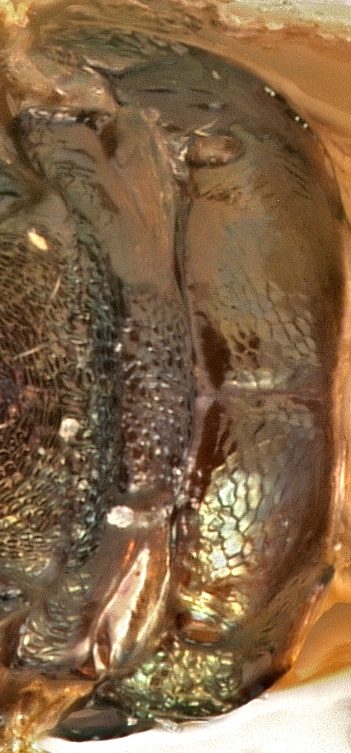

Supplement: Supplementary material 2 — LUCID Key for Conidarnes [file zookeys-539-119-s002.zip › Conidarnes_Key_lucid/Key to Species of Conidarnes Farache & Rasplus/Media/Images/11_propodeum_sulcus_depressed.jpg]

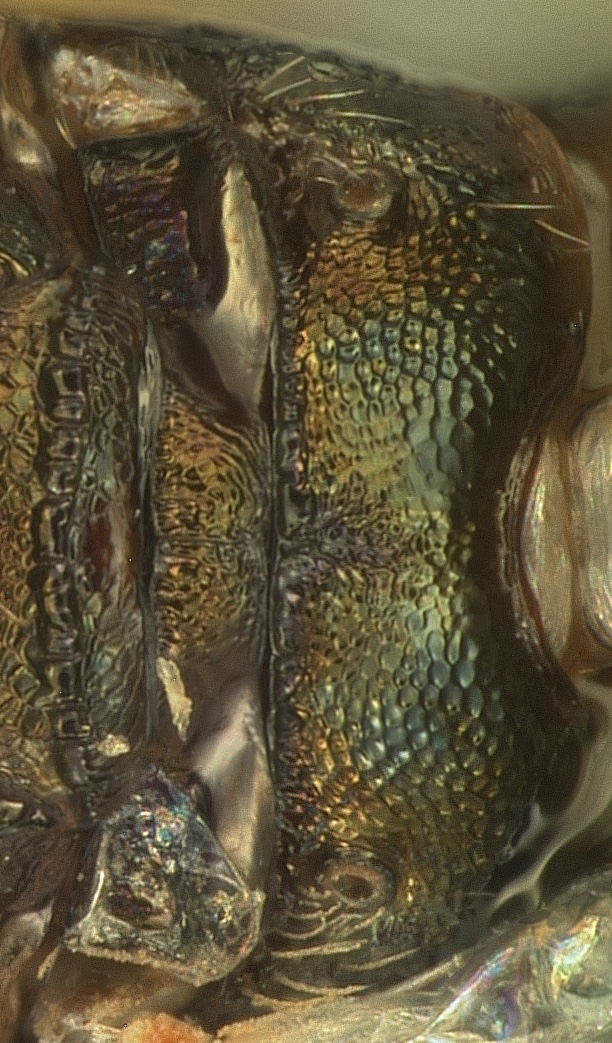

Supplement: Supplementary material 2 — LUCID Key for Conidarnes [file zookeys-539-119-s002.zip › Conidarnes_Key_lucid/Key to Species of Conidarnes Farache & Rasplus/Media/Images/11_propodeum_sulcus_striated.jpg]

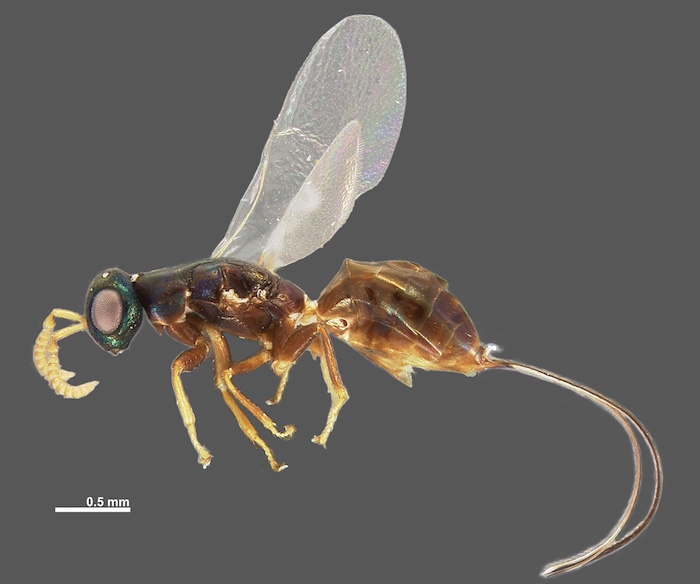

Supplement: Supplementary material 2 — LUCID Key for Conidarnes [file zookeys-539-119-s002.zip › Conidarnes_Key_lucid/Key to Species of Conidarnes Farache & Rasplus/Media/Images/12_0vip_1.0.jpg]

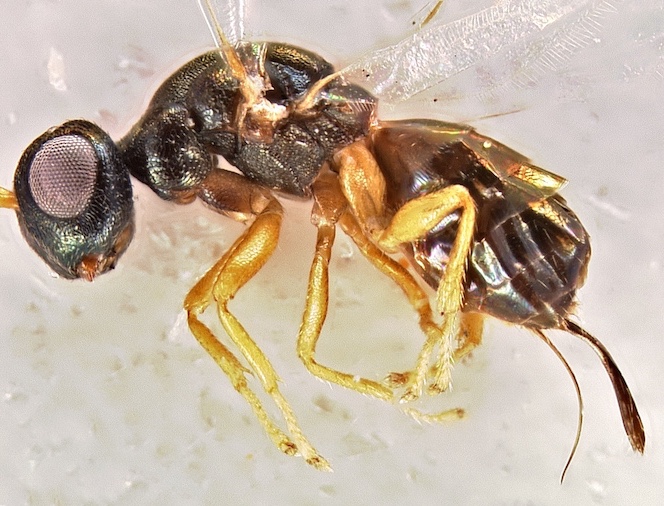

Supplement: Supplementary material 2 — LUCID Key for Conidarnes [file zookeys-539-119-s002.zip › Conidarnes_Key_lucid/Key to Species of Conidarnes Farache & Rasplus/Media/Images/12_ovip_0.5.jpg]

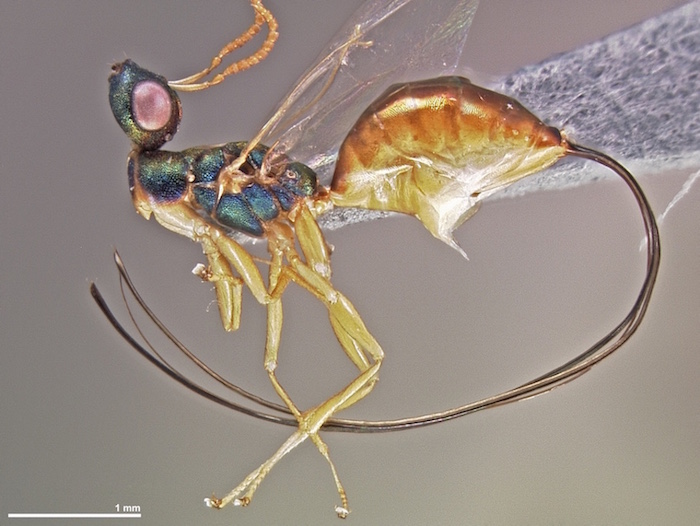

Supplement: Supplementary material 2 — LUCID Key for Conidarnes [file zookeys-539-119-s002.zip › Conidarnes_Key_lucid/Key to Species of Conidarnes Farache & Rasplus/Media/Images/12_ovip_1.5.jpg]

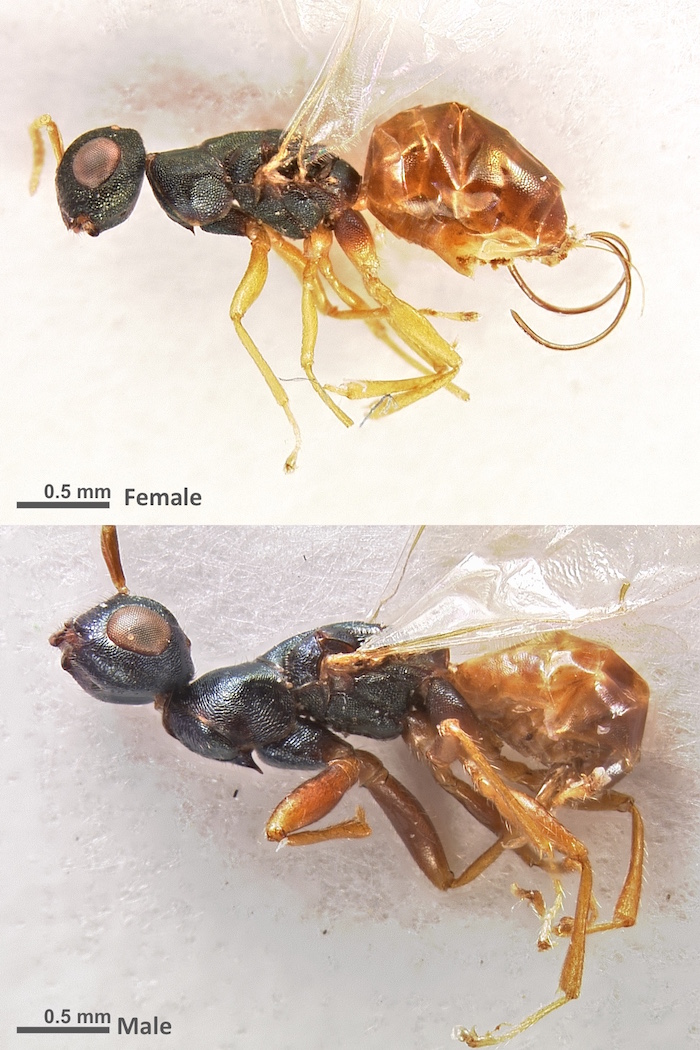

Supplement: Supplementary material 2 — LUCID Key for Conidarnes [file zookeys-539-119-s002.zip › Conidarnes_Key_lucid/Key to Species of Conidarnes Farache & Rasplus/Media/Images/hab_bergi.jpg]

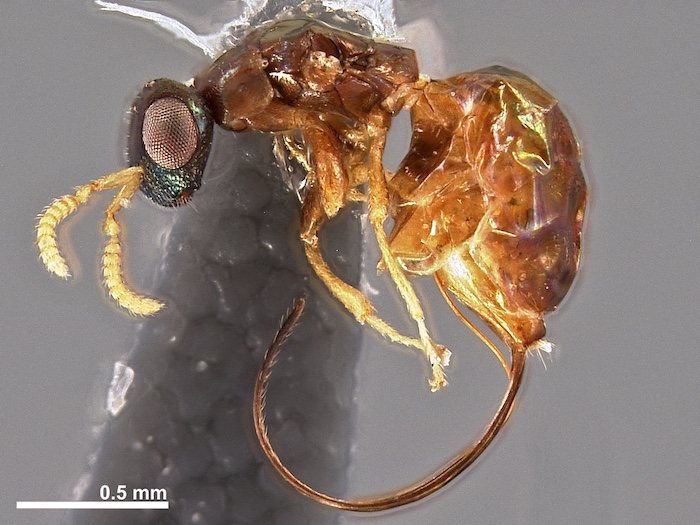

Supplement: Supplementary material 2 — LUCID Key for Conidarnes [file zookeys-539-119-s002.zip › Conidarnes_Key_lucid/Key to Species of Conidarnes Farache & Rasplus/Media/Images/hab_laevis.jpg]

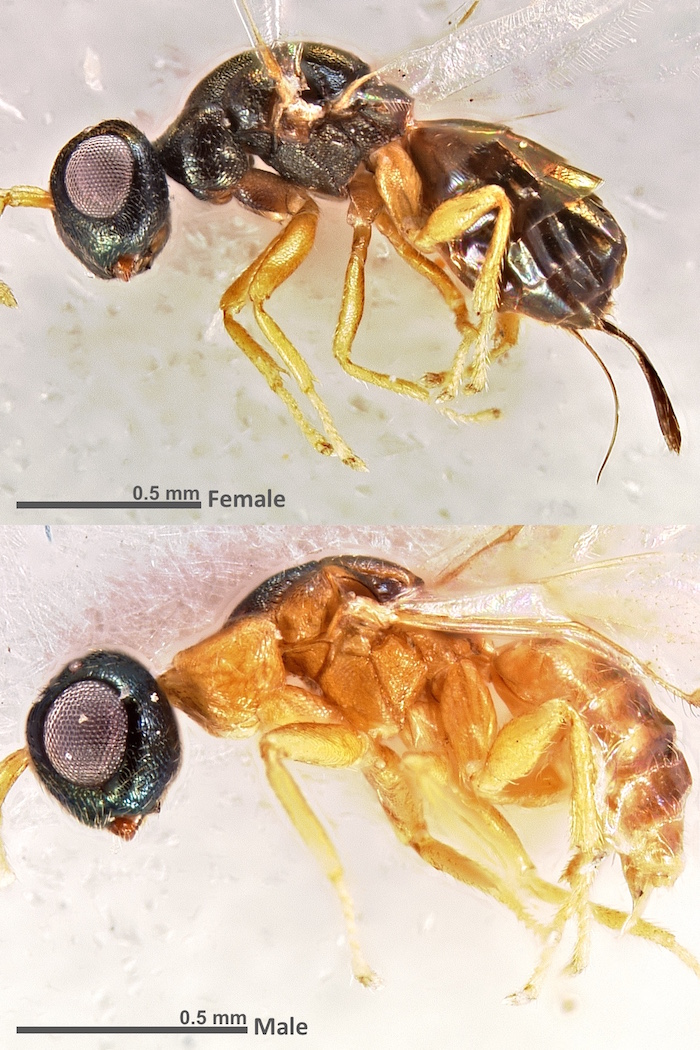

Supplement: Supplementary material 2 — LUCID Key for Conidarnes [file zookeys-539-119-s002.zip › Conidarnes_Key_lucid/Key to Species of Conidarnes Farache & Rasplus/Media/Images/hab_santineloi.jpg]

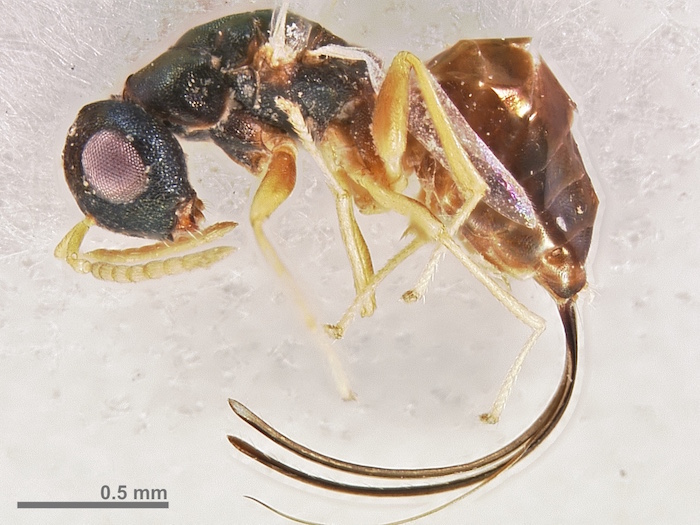

Supplement: Supplementary material 2 — LUCID Key for Conidarnes [file zookeys-539-119-s002.zip › Conidarnes_Key_lucid/Key to Species of Conidarnes Farache & Rasplus/Media/Images/hab_subtectae.jpg]

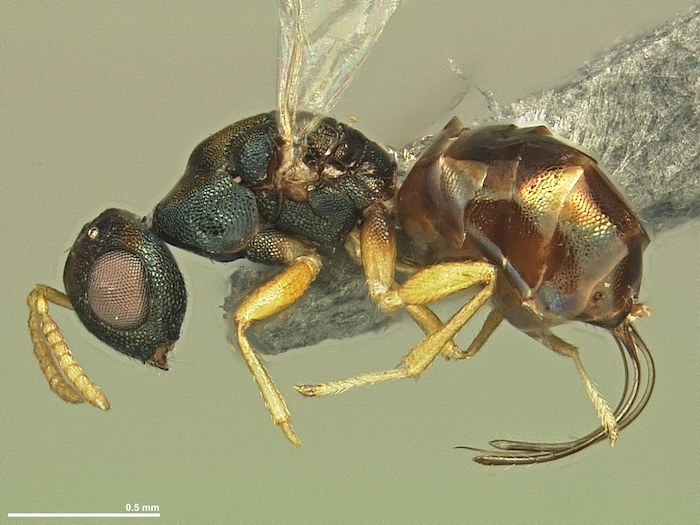

Supplement: Supplementary material 2 — LUCID Key for Conidarnes [file zookeys-539-119-s002.zip › Conidarnes_Key_lucid/Key to Species of Conidarnes Farache & Rasplus/Media/Images/hab_sumatranae.jpg]

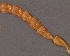

Supplement: Supplementary material 2 — LUCID Key for Conidarnes [file zookeys-539-119-s002.zip › Conidarnes_Key_lucid/Key to Species of Conidarnes Farache & Rasplus/Media/Thumbs/01_funicular_long_TN.jpg]

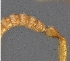

Supplement: Supplementary material 2 — LUCID Key for Conidarnes [file zookeys-539-119-s002.zip › Conidarnes_Key_lucid/Key to Species of Conidarnes Farache & Rasplus/Media/Thumbs/01_funicular_short_TN.jpg]

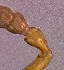

Supplement: Supplementary material 2 — LUCID Key for Conidarnes [file zookeys-539-119-s002.zip › Conidarnes_Key_lucid/Key to Species of Conidarnes Farache & Rasplus/Media/Thumbs/02_anelli_1_TN.jpg]

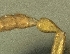

Supplement: Supplementary material 2 — LUCID Key for Conidarnes [file zookeys-539-119-s002.zip › Conidarnes_Key_lucid/Key to Species of Conidarnes Farache & Rasplus/Media/Thumbs/02_anelli_2_TN.jpg]

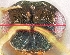

Supplement: Supplementary material 2 — LUCID Key for Conidarnes [file zookeys-539-119-s002.zip › Conidarnes_Key_lucid/Key to Species of Conidarnes Farache & Rasplus/Media/Thumbs/03_insertion below middle_TN.jpg]

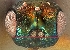

Supplement: Supplementary material 2 — LUCID Key for Conidarnes [file zookeys-539-119-s002.zip › Conidarnes_Key_lucid/Key to Species of Conidarnes Farache & Rasplus/Media/Thumbs/03_insertion lower_TN.jpg]

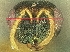

Supplement: Supplementary material 2 — LUCID Key for Conidarnes [file zookeys-539-119-s002.zip › Conidarnes_Key_lucid/Key to Species of Conidarnes Farache & Rasplus/Media/Thumbs/03_insertion middle_TN.jpg]

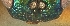

Supplement: Supplementary material 2 — LUCID Key for Conidarnes [file zookeys-539-119-s002.zip › Conidarnes_Key_lucid/Key to Species of Conidarnes Farache & Rasplus/Media/Thumbs/04_supraclyp_inconspicuous_TN.jpg]

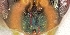

Supplement: Supplementary material 2 — LUCID Key for Conidarnes [file zookeys-539-119-s002.zip › Conidarnes_Key_lucid/Key to Species of Conidarnes Farache & Rasplus/Media/Thumbs/04_supraclyp_longer_TN.jpg]

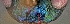

Supplement: Supplementary material 2 — LUCID Key for Conidarnes [file zookeys-539-119-s002.zip › Conidarnes_Key_lucid/Key to Species of Conidarnes Farache & Rasplus/Media/Thumbs/04_supraclyp_shorter_TN.jpg]

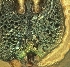

Supplement: Supplementary material 2 — LUCID Key for Conidarnes [file zookeys-539-119-s002.zip › Conidarnes_Key_lucid/Key to Species of Conidarnes Farache & Rasplus/Media/Thumbs/05_supraclyp_narrow_TN.jpg]

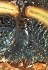

Supplement: Supplementary material 2 — LUCID Key for Conidarnes [file zookeys-539-119-s002.zip › Conidarnes_Key_lucid/Key to Species of Conidarnes Farache & Rasplus/Media/Thumbs/05_supraclyp_wide_TN.jpg]

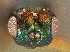

Supplement: Supplementary material 2 — LUCID Key for Conidarnes [file zookeys-539-119-s002.zip › Conidarnes_Key_lucid/Key to Species of Conidarnes Farache & Rasplus/Media/Thumbs/06_face_reticulate_scrobe smooth_TN.jpg]

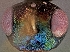

Supplement: Supplementary material 2 — LUCID Key for Conidarnes [file zookeys-539-119-s002.zip › Conidarnes_Key_lucid/Key to Species of Conidarnes Farache & Rasplus/Media/Thumbs/06_face_reticulate_TN.jpg]

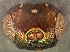

Supplement: Supplementary material 2 — LUCID Key for Conidarnes [file zookeys-539-119-s002.zip › Conidarnes_Key_lucid/Key to Species of Conidarnes Farache & Rasplus/Media/Thumbs/06_face_upper smooth_TN.jpg]

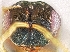

Supplement: Supplementary material 2 — LUCID Key for Conidarnes [file zookeys-539-119-s002.zip › Conidarnes_Key_lucid/Key to Species of Conidarnes Farache & Rasplus/Media/Thumbs/07_scrobal_sulcus_absent_TN.jpg]

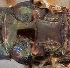

Supplement: Supplementary material 2 — LUCID Key for Conidarnes [file zookeys-539-119-s002.zip › Conidarnes_Key_lucid/Key to Species of Conidarnes Farache & Rasplus/Media/Thumbs/08_mesoscutum_laterally engraved_TN.jpg]

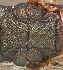

Supplement: Supplementary material 2 — LUCID Key for Conidarnes [file zookeys-539-119-s002.zip › Conidarnes_Key_lucid/Key to Species of Conidarnes Farache & Rasplus/Media/Thumbs/08_mesoscutum_reticulate_TN.jpg]

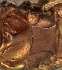

Supplement: Supplementary material 2 — LUCID Key for Conidarnes [file zookeys-539-119-s002.zip › Conidarnes_Key_lucid/Key to Species of Conidarnes Farache & Rasplus/Media/Thumbs/08_mesoscutum_smooth_TN.jpg]

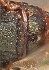

Supplement: Supplementary material 2 — LUCID Key for Conidarnes [file zookeys-539-119-s002.zip › Conidarnes_Key_lucid/Key to Species of Conidarnes Farache & Rasplus/Media/Thumbs/10_metascutellum_inconspicuous_TN.jpg]

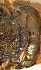

Supplement: Supplementary material 2 — LUCID Key for Conidarnes [file zookeys-539-119-s002.zip › Conidarnes_Key_lucid/Key to Species of Conidarnes Farache & Rasplus/Media/Thumbs/10_metascutellum_long_TN.jpg]

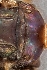

Supplement: Supplementary material 2 — LUCID Key for Conidarnes [file zookeys-539-119-s002.zip › Conidarnes_Key_lucid/Key to Species of Conidarnes Farache & Rasplus/Media/Thumbs/10_metascutellum_smooth_TN.jpg]

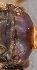

Supplement: Supplementary material 2 — LUCID Key for Conidarnes [file zookeys-539-119-s002.zip › Conidarnes_Key_lucid/Key to Species of Conidarnes Farache & Rasplus/Media/Thumbs/11_propodeum_sulcus_absent_TN.jpg]

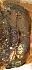

Supplement: Supplementary material 2 — LUCID Key for Conidarnes [file zookeys-539-119-s002.zip › Conidarnes_Key_lucid/Key to Species of Conidarnes Farache & Rasplus/Media/Thumbs/11_propodeum_sulcus_depressed_TN.jpg]

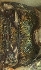

Supplement: Supplementary material 2 — LUCID Key for Conidarnes [file zookeys-539-119-s002.zip › Conidarnes_Key_lucid/Key to Species of Conidarnes Farache & Rasplus/Media/Thumbs/11_propodeum_sulcus_striated_TN.jpg]

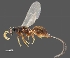

Supplement: Supplementary material 2 — LUCID Key for Conidarnes [file zookeys-539-119-s002.zip › Conidarnes_Key_lucid/Key to Species of Conidarnes Farache & Rasplus/Media/Thumbs/12_0vip_1.0_TN.jpg]

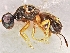

Supplement: Supplementary material 2 — LUCID Key for Conidarnes [file zookeys-539-119-s002.zip › Conidarnes_Key_lucid/Key to Species of Conidarnes Farache & Rasplus/Media/Thumbs/12_ovip_0.5_TN.jpg]

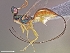

Supplement: Supplementary material 2 — LUCID Key for Conidarnes [file zookeys-539-119-s002.zip › Conidarnes_Key_lucid/Key to Species of Conidarnes Farache & Rasplus/Media/Thumbs/12_ovip_1.5_TN.jpg]

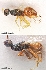

Supplement: Supplementary material 2 — LUCID Key for Conidarnes [file zookeys-539-119-s002.zip › Conidarnes_Key_lucid/Key to Species of Conidarnes Farache & Rasplus/Media/Thumbs/hab_bergi_TN.jpg]

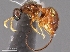

Supplement: Supplementary material 2 — LUCID Key for Conidarnes [file zookeys-539-119-s002.zip › Conidarnes_Key_lucid/Key to Species of Conidarnes Farache & Rasplus/Media/Thumbs/hab_laevis_TN.jpg]

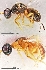

Supplement: Supplementary material 2 — LUCID Key for Conidarnes [file zookeys-539-119-s002.zip › Conidarnes_Key_lucid/Key to Species of Conidarnes Farache & Rasplus/Media/Thumbs/hab_santineloi_TN.jpg]

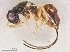

Supplement: Supplementary material 2 — LUCID Key for Conidarnes [file zookeys-539-119-s002.zip › Conidarnes_Key_lucid/Key to Species of Conidarnes Farache & Rasplus/Media/Thumbs/hab_subtectae_TN.jpg]

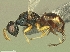

Supplement: Supplementary material 2 — LUCID Key for Conidarnes [file zookeys-539-119-s002.zip › Conidarnes_Key_lucid/Key to Species of Conidarnes Farache & Rasplus/Media/Thumbs/hab_sumatranae_TN.jpg]
